# Comparative Efficacy and Safety of Ecnoglutide in Type 2 Diabetes: A Systematic Review and Meta‐Analysis

**DOI:** 10.1002/edm2.70217

**Published:** 2026-04-05

**Authors:** Mukesh Kumar, Sumet Kumar, Sheikh Muhammad Ebtehaj Ali, Hadia Qazi, Arshia Arif Janjua, Hanzlah Ahmed, Rayan Babar, Rumaisa Imtiaz Sheikh, Laksh Kumar, Sana Mohammad Ali, Fatima Hanif, Khizra Gul, Ayesha Sarwar Ali, Shehzadi Umme Rubab Bukhari, Adarsh Raja, Aayush Chaulagain

**Affiliations:** ^1^ Department of Medicine Shaheed Mohtarma Benazir Bhutto Medical College Lyari Karachi Pakistan; ^2^ Department of Medicine, Abbasi Shaheed Hospital Karachi Pakistan; ^3^ Department of Medicine Patan Academy of Health Sciences Lalitpur Nepal

**Keywords:** Ecnoglutide, GLP‐1 receptor agonist, glycaemic control, meta‐analysis, type 2 diabetes mellitus

## Abstract

**Background:**

Type 2 diabetes mellitus (T2DM) affects over 500 million people worldwide, with traditional therapies often failing to maintain long‐term glycaemic control. Ecnoglutide, a novel long‐acting GLP‐1 receptor agonist, has emerged as a promising therapeutic option. This systematic review and meta‐analysis evaluated the efficacy and safety of ecnoglutide in adults with T2DM.

**Methods:**

Following PRISMA guidelines, we systematically searched PubMed, Cochrane Library, ScienceDirect, ClinicalTrials.gov, and Google Scholar through September 2025. Randomized controlled trials comparing ecnoglutide with placebo or active comparators in adults with T2DM were included. Primary outcomes were changes in HbA1c and body weight. Secondary outcomes included fasting plasma glucose, insulin resistance markers, lipid profile, liver enzymes, and adverse events. Risk of bias was assessed using the Cochrane RoB‐2 tool. Meta‐analysis was performed using random‐effects models, with mean differences and risk ratios calculated at 95% confidence intervals.

**Results:**

Four RCTs comprising 1643 participants (1162 receiving ecnoglutide, 444 controls) were included. Ecnoglutide significantly reduced HbA1c (MD = −0.44, 95% CI −0.55 to −0.33, *p* < 0.00001), body weight (MD = −5.63, 95% CI −7.90 to −3.35, *p* < 0.01), and fasting plasma glucose (MD = −0.81, 95% CI −1.03 to −0.59, *p* < 0.00001). Improvements were observed in insulin sensitivity, lipid profile, and liver enzymes. Adverse events occurred more frequently with ecnoglutide (RR = 1.09, *p* < 0.01), although predominantly gastrointestinal and mild‐to‐moderate, with no significant differences in serious adverse events.

**Conclusions:**

Ecnoglutide demonstrates robust efficacy in glycaemic control, weight reduction, and cardiometabolic parameters with an acceptable safety profile in adults with T2DM, supporting its therapeutic potential as a next‐generation GLP‐1 receptor agonist.

## Graphical abstract

Ecnoglutide, a once‐weekly GLP‐1 receptor agonist, significantly improves glycaemic control and promotes meaningful weight loss compared with placebo in type 2 diabetes. This systematic review and meta‐analysis of four RCTs demonstrates favourable efficacy with mostly mild gastrointestinal adverse events and no increase in serious safety concerns.

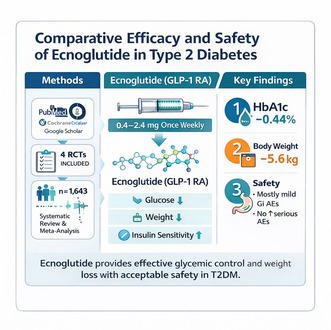

## 1 Inroduction

More than 500 million individuals are affected by diabetes mellitus worldwide, resulting in 6.7 million deaths annually by chronic kidney diseases, cardiovascular diseases, and microvascular complications [1]. Nationwide analysis of the United States highlights diabetes mellitus as a leading cause of renal failure, cardiovascular morbidity, blindness, lower limb amputations, and microvascular complications [2]. Insulin resistance, β‐cell dysfunction, and impaired glucose regulation are the hallmarks of type 2 diabetes mellitus (T2DM), a chronic and progressive metabolic disease [3]. Despite advancements in pharmacotherapy, T2DM individuals fail to achieve controlled glycaemic status and significantly experience treatment‐related side effects [3, 4]. T2DM is one of the most important non‐communicable health issues in the world, and its burden has increased due to the rising prevalence of obesity, sedentary lifestyles, and poor eating habits [2].

The traditional treatment of type 2 diabetes involves pharmacological therapy in conjunction with lifestyle changes like diet, exercise, and weight control. Metformin, sulphonylureas, DPP‐4 inhibitors, SGLT2 inhibitors, and insulin are examples of common glucose‐lowering medications [5, 6]. Despite their effectiveness in lowering blood glucose, these medications frequently do not preserve long‐term glycaemic stability and can result in adverse effects such as weight gain, hypoglycaemia, and gastrointestinal intolerance [7]. As a result, there is a growing demand for newer medications with long‐lasting effectiveness, weight benefits, and advantageous cardiometabolic profiles [8].

By treating the glycaemic and metabolic components of type 2 diabetes, glucagon‐like peptide‐1 receptor agonists, or GLP‐1 RAs, have revolutionized the treatment of diabetes. They improve cardiovascular outcomes while promoting notable decreases in HbA1c and body weight through glucose‐dependent insulin release, glucagon suppression, delayed gastric emptying, and appetite regulation [9, 10, 11].

The next development in this therapeutic class is Ecnoglutide, a long‐acting novel GLP‐1 RA. According to the recent randomized controlled trials, significantly lowers body weight, insulin resistance indices, fasting plasma glucose, and HbA1c. It also has positive effects on lipid and liver profiles [12, 13]. Compared with previous GLP‐1 analogues, its longer half‐life and once‐weekly dosage may improve adherence and tolerability. Moreover, due to the long‐acting nature of Ecnoglutide with distinct pharmacodynamic properties, it was selected as a novel intervention for this insightful review. Prolonged half‐life, enhanced resistance to dipeptidyl peptidase degradation and prominently improving selectivity of receptors from clinical data signifies the role of ecnoglutide, over other established GLP‐1 RAs like semaglutide and dulaglutide [13]. Phase 2 and phase 3 trials of ecnoglutide, as an investigational agent, provide growing trial evidence of the clinical efficacy of this promising intervention.

In light of these encouraging results, the purpose of this systematic review and meta‐analysis is to thoroughly assess the placebo effectiveness of Ecnoglutide in adults with type 2 diabetes mellitus by contrasting it with active treatments or a placebo. To ascertain its overall therapeutic potential in the contemporary management of type 2 diabetes, the analysis focuses on the primary outcomes of HbA1c and body weight, as well as the secondary outcomes of fasting plasma glucose, insulin resistance, lipid profile, liver enzymes, and adverse events.

## 2 Methods

We conducted this systematic review and meta‐analysis in accordance with AMSTAR 2 tool and PRISMA guidelines, adhering to established standards for the rigorous conduct and transparent reporting of research syntheses [14]. Applying these guidelines ensured clear documentation of our methods and strengthened the reliability and reproducibility of our findings, ultimately supporting the overall credibility of our conclusions. As per the TITAN Guidelines 2025 governing transparent AI implementation in scholarly publications, AI assistance was confined exclusively to linguistic enhancement during manuscript development, with no involvement in designing the study, collecting data, performing analyses, or interpreting results [15]. The review protocol is available in the PROSPERO database under registration number CRD420261284992.

### 2.1 Search Strategy and Study Selection

A comprehensive search was carried out in PubMed, Cochrane Library, ScienceDirect, ClinicalTrials.gov, and Google Scholar up to September 2025. The search terms used were “Ecnoglutide,” “GLP‐1 receptor agonist,” “type 2 diabetes,” “HbA1c,” and “randomized controlled trial.” Reference lists of related reviews and included studies were also checked to identify additional eligible trials (Table S1).

The study selection followed the PRISMA framework. All search results were imported into EndNote X7.5 to remove duplicates. Two reviewers independently screened titles and abstracts, followed by full‐text reviews to identify eligible studies. Disagreements were resolved through discussion or consultation with a third reviewer.

Inclusion criteria were randomized controlled trials (RCTs) involving adults (≥ 18 years) with type 2 diabetes mellitus (T2DM), comparing Ecnoglutide with either placebo or active comparators, and reporting at least one measurable outcome related to glycaemic or metabolic control such as HbA1c, fasting plasma glucose (FPG), insulin resistance, body weight, or adverse events.

Exclusion criteria included non‐randomized or observational studies, preclinical or single‐arm studies, and trials lacking comparative quantitative outcomes. Narrative reviews, editorials, and conference abstracts were also excluded.

### 2.2 Data Extraction

Data extraction was performed using a standardized Excel sheet. Extracted data included study name, publication year, clinical trial registration number, sample size, participant characteristics (age, BMI, HbA1c, diabetes duration), intervention and comparator details, dose and treatment duration, and reported outcomes. The main outcomes assessed included measures of glycaemic and insulin regulation, such as glycated haemoglobin (HbA1c), fasting plasma glucose (FPG), 2‐h post‐prandial blood glucose, seven‐point self‐monitored blood glucose (SMBG), fasting insulin, and the homeostatic model assessment of insulin resistance (HOMA‐IR). Additional outcomes covered anthropometric measures including body weight, body mass index (BMI), and waist circumference (WC), as well as lipid profile parameters such as total cholesterol, low‐density lipoprotein cholesterol (LDL‐C), triglycerides (TG), and high‐density lipoprotein cholesterol (HDL‐C). Liver function was evaluated using alanine aminotransferase (ALT) and aspartate aminotransferase (AST). Safety assessments included any adverse events, serious adverse events, treatment‐related serious adverse events, and adverse events leading to treatment discontinuation. Two reviewers conducted the extraction independently, and any differences were resolved by consensus.

### 2.3 Risk of Bias Assessment

The quality of the included studies was assessed using the Cochrane Risk of Bias Tool (RoB‐2) [16]. Each trial was evaluated for randomization, blinding, allocation concealment, completeness of outcome data, and selective reporting. Two reviewers independently assessed bias risk, and disagreements were resolved through discussion.

### 2.4 Certainty of Evidence (GRADE Assessment)

The certainty of evidence for key outcomes was evaluated using the **Grading of Recommendations Assessment, Development and Evaluation (GRADE)** approach. As all included studies were RCTs, evidence initially started at high certainty and was downgraded based on predefined criteria, including risk of bias, inconsistency, indirectness, imprecision, and publication bias. GRADE assessments were performed independently by two reviewers, and summary judgements were presented in the RCTs; evidence initially started at high certainty and was downgraded based on predefined criteria, including risk of bias, inconsistency, indirectness, imprecision, and publication bias [17].

### 2.5 Statistical Analysis

Statistical analysis was conducted using Review Manager (RevMan 5.4). Continuous outcomes were presented as mean differences (MDs) with 95% confidence intervals (CIs), while dichotomous outcomes were expressed as risk ratios (RRs) with 95% CIs. We pooled different doses of ecnoglutide in the primary analysis to estimate the overall class‐level efficacy of ecnoglutide compared with control, consistent with previous meta‐analyses of incretin‐based therapies [18, 19]. A random‐effects model was applied to account for variability among studies. Heterogeneity was assessed using the I^2^ statistic, with values above 50% indicating significant heterogeneity [20]. Sensitivity analysis was performed by excluding one study at a time to check the robustness of results. Subgroup analyses were conducted to explore potential sources of heterogeneity. These were performed on the dosage categories used across studies (0.4 mg, 0.6 mg, 0.8 mg, 1.2 mg, 1.8 mg, and 2.4 mg) and on follow‐up duration, which was classified as short‐term (20–24 weeks), intermediate‐term (32–40 weeks), and long‐term (48–52 weeks). A *p*‐value of less than 0.05 was considered statistically significant. Due to the limited number of trials per individual dose, all available doses were pooled in the primary analysis to enhance statistical power, with dose‐stratified subgroup analyses conducted to explore potential dose–response effects.

## 3 Results

### 3.1 Study Selection and Characteristics

**FIGURE 1:**
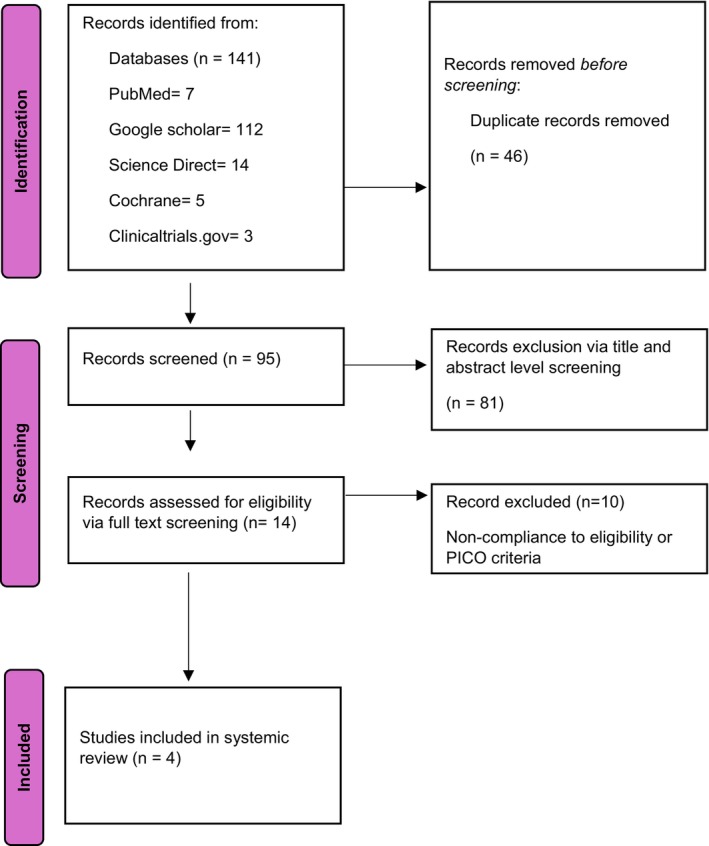
PRISMA flow chart.

The systematic literature search identified 141 records across five databases: Google Scholar (*n* = 112), Science Direct (*n* = 14), PubMed (*n* = 7), Cochrane Library (*n* = 5), and ClinicalTrials.gov (*n* = 3). After removing 46 duplicates, 95 unique records underwent title and abstract screening, of which 81 were excluded for not meeting inclusion criteria. Full‐text assessment of 14 articles resulted in the exclusion of 10 articles due to non‐compliance with eligibility or PICO criteria. Ultimately, 4 RCTs were included in the meta‐analysis. The selection process is illustrated in the PRISMA flow diagram (Figure 1).

The included trials were published between 2023 and 2025, comprising a total of 1643 participants (1162 receiving ecnoglutide and 444 receiving placebo or active comparator). Three studies were phase 3 trials, and one was a phase 2 trial. Dalong et al. 2023 was a multicentre, double‐blind, placebo‐controlled phase 2 trial; Yang et al. 2025 was an open‐label, active‐controlled phase 3 trial (EECOH‐2); Linong et al. 2025 was a multicentre, double‐blind, placebo‐controlled phase 3 trial (SLIMMER); and Bing et al. 2025 was a double‐blind, placebo‐controlled phase 3 trial. The mean age of participants ranged from 34 to 54 years across treatment groups, with males comprising 50%–68% of participants. Mean baseline HbA1c ranged from 5.3% to 8.67%, and mean BMI ranged from 25.8 to 32.9 kg/m^2^. Ecnoglutide was administered subcutaneously at doses ranging from 0.4 mg to 2.4 mg. The primary outcomes included change in HbA1c from baseline and percentage change in bodyweight. Detailed study characteristics are presented in Tables 1 and 2.

**TABLE 1:** Study characteristics.

Study	Clinical trial no	Patient category	Dose	Total sample	Sample size		Study design	Primary outcome
					Ecnoglutide	Control		
Dalong et al. 2023	CTR20211014	The ecnoglutide group had a mean age of 50.2 years, a higher proportion of males (67.9%), and a longer diabetes duration (49.7 months) compared with the placebo group (49.7 years, 52.8% male, 40.4 months).	0.4 mg, 0.8 mg, 1.2 mg	145	109	36	This completed Phase 2, multicenter, randomized, double‐blind, placebo‐controlled clinical trial	HbA1c levels declined for all ecnoglutide dose groups over the course of treatment
Yang et al. 2025	NCT05680129	Eligible participants were adults aged 18–75 years with BMI 20–35 kg/m^2^, diagnosed with type 2 diabetes for ≥ 3 months, and on stable metformin monotherapy (≥ 1500 mg/day or ≥ 1000 mg/day if intolerant) plus diet and exercise for the 8 weeks before screening.	0.6 mg, 1.2 mg	623	414	207	EECOH‐2 was an open‐label, active‐controlled, randomized, phase 3 trial	The primary efficacy endpoint was the change from baseline in HbA1c to week 32.
Linong et al. 2025	NCT05813795	Eligible adults were 18–75 years old with BMI ≥ 28 kg/m^2^, or ≥ 24 kg/m^2^ with multiple weight‐related comorbidities and had ≤ 5% self‐reported weight loss with diet and exercise in the 3 months before screening.	1.2 mg, 1.8 mg, 2.4 mg	664	499	165	SLIMMER was a multicentre, randomized, double blind, placebo‐controlled, phase 3 trial	The coprimary efficacy endpoints were the percentage change in bodyweight from baseline and the proportion of participants with reduction of 5% or more in bodyweight at week 40.
Bing et al. 2025	NCT05680155	Mean (SD) age was 52.0 (10.9) years, 60.2% were male, with mean (SD) HbA1c 8.52% (0.81), BMI 26.93 kg/m^2^ (3.40), and T2DM duration 3.58 (3.78) years.	0.6 mg, 1.2 mg	211	140	36	arandomized, double‐blind, placebo‐controlled, phase 3 trial	The primary endpoint was met, and both doses of ecnoglutide signi cantly reduced HbA1c more than placebo for both primary and secondary e cacy estimands.

**TABLE 2:** Baseline characteristics.

Characteristics	Dalong et al. 2023				Yang et al. 2025			Linong et al. 2025				Bing et al. 2025		
	Ecnoglutide 0.4 mg	Ecnoglutide 0.8 mg	Ecnoglutide 1.2 mg	Placebo	Ecnoglutide 1.2 mg	Ecnoglutide 0.6 mg	Dulaglutide	Ecnoglutide 1.2 mg	Ecnoglutide 1.8 mg	Ecnoglutide 2.4 mg	Placebo	Ecnoglutide 1.2 mg	Ecnoglutide 0.6 mg	Placebo
Age (years)	49.1 (8.87)	51.8 (10.34)	49.6 (9.65)	49.7 (10.54)	54.1 (10.11)	54.2 (10.89)	53.4 (9.28)	34.0 (7.3)	34.1 (7.7)	35.0 (7.9)	33.8 (7.2)	52.5 (11.5)	52.1 (10.8)	51.4 (10.4)
Male	25 (67.6)	28 (77.8)	21 (58.3)	19 (52.8)	113 (54%)	120 (58%)	114 (55%)	83 (50%)	76 (46%)	94 (56%)	82 (50%)	43 (61%)	39 (57%)	45 (63%)
Female	12 (32.4)	8 (22.2)	15 (41.7)	17 (47.2)	95 (46%)	86 (42%)	93 (45%)	83 (50%)	90 (54%)	73 (44%)	83 (50%)	28 (39%)	30 (43%)	26 (37%)
Body weight (kg)	72.8 (14.6)	76.6 (13.4)	72.5 (12.1)	71.8 (12.2)	74.22 (12.27)	73.73 (12.90)	72.77 (14.15)	92.2 (17.4)	91.4 (14.9)	90.6 (16.0)	91.0 (16.3)	71.53 (12.05)	74.25 (15.20)	73.69 (11.31)
BMI (kg/m2)	25.8 (3.7)	26.6 (3.4)	26.2 (3.0)	26.4 (3.2)	27.22 (3.59)	26.87 (3.45)	26.64 (3.65)	32.8 (4.4)	32.9 (4.3)	31.9 (3.6)	32.4 (4.1)	26.35 (3.31)	27.23 (3.65)	27.23 (3.20)
Waist circumference, cm	N/A	N/A	N/A	N/A	95.97 (9.18)	95.56 (9.56)	94.44 (10.32)	104.4 (11.8)	104.4 (10.8)	103.2 (9.9)	104.0 (10.4)	93.26 (8.94)	94.02 (10.55)	94.60 (8.36)
HbA1c (%)	8.45 (0.64)	8.65 (0.76)	8.67 (0.75)	8.44 (0.67)	8.41% (0.78)	8.39% (0.79)	8.40% (0.78)	5.3 (0.3)	5.4 (0.3)	5.3 (0.3)	5.3 (0.4)	8.51 (0.83)	8.54 (0.80)	8.51 (0.81)
HbA1c, mmol/mol	N/A	N/A	N/A	N/A	68.40 (8.47)	68.15 (8.62)	68.28 (8.56)	34.6 (3.4)	35.0 (3.8)	34.2 (3.8)	34.2 (4.1)	69.51 (9.10)	69.84 (8.72)	69.51 (8.89)
FPG (mmol/L)	9.70 (1.605)	11.08 (1.678)	10.00 (1.889)	10.50 (1.800)	9.56 (1.82)	9.50 (1.95)	9.51 (1.93)	5.3 (0.4)	5.3 (0.5)	5.3 (0.5)	5.2 (0.5)	9.67 (1.86)	9.67 (1.76)	9.81 (1.82)
Fasting insulin, μU/mL	N/A	N/A	N/A	N/A	9.40 (6.32–15.19)	9.62 (5.82–15.80)	9.18 (5.46–14.73)	22.7 (14.8)	21.4 (12.8)	21.4 (13.5)	20.7 (10.5)	N/A	N/A	N/A
Diabetes duration (months)	46.23 (51.93)	57.64 (44.18)	45.39 (49.55)	40.36 (35.58)	N/A	N/A	N/A	N/A	N/A	N/A	N/A	N/A	N/A	N/A
Diabetes duration, years	N/A	N/A	N/A	N/A	6.35 (3.30–10.20)	5.40 (3.30–9.50)	5.30 (3.30–9.90)	N/A	N/A	N/A	N/A	3.66 (3.99)	4.10 (4.20)	3.00 (3.02)
Previous antihyperglycemic treatment, *n* (%)	15 (40.5)	23 (63.9)	22 (61.1)	19 (52.8)	N/A	N/A	N/A	N/A	N/A	N/A	N/A	35 (49)	41 (59)	26 (37)
eGFR, mL/min per 1.73 m2	N/A	N/A	N/A	N/A	119.431 (27.27)	126.081 (35.79)	121.77 (29.45)	103.4 (16.2)	104.3 (17.5)	104.0 (16.9)	104.4 (18.0)	124.7 (35.1)	122.0 (29.9)	113.5 (25.9)
Systolic blood pressure, mm Hg	N/A	N/A	N/A	N/A	125.6 (11.57)	125.0 (11.76)	125.4 (11.43)	121.1 (11.1)	120.8 (12.0)	122.7 (12.1)	120.6 (10.6)	N/A	N/A	N/A
Diastolic blood pressure, mm Hg	N/A	N/A	N/A	N/A	81.9 (7.92)	81.4 (7.71)	82.0 (7.63)	83.0 (8.0)	82.5 (8.5)	82.5 (8.3)	82.7 (7.9)	N/A	N/A	N/A

### 3.2 Risk of Bias Assessment

**FIGURE 2:**
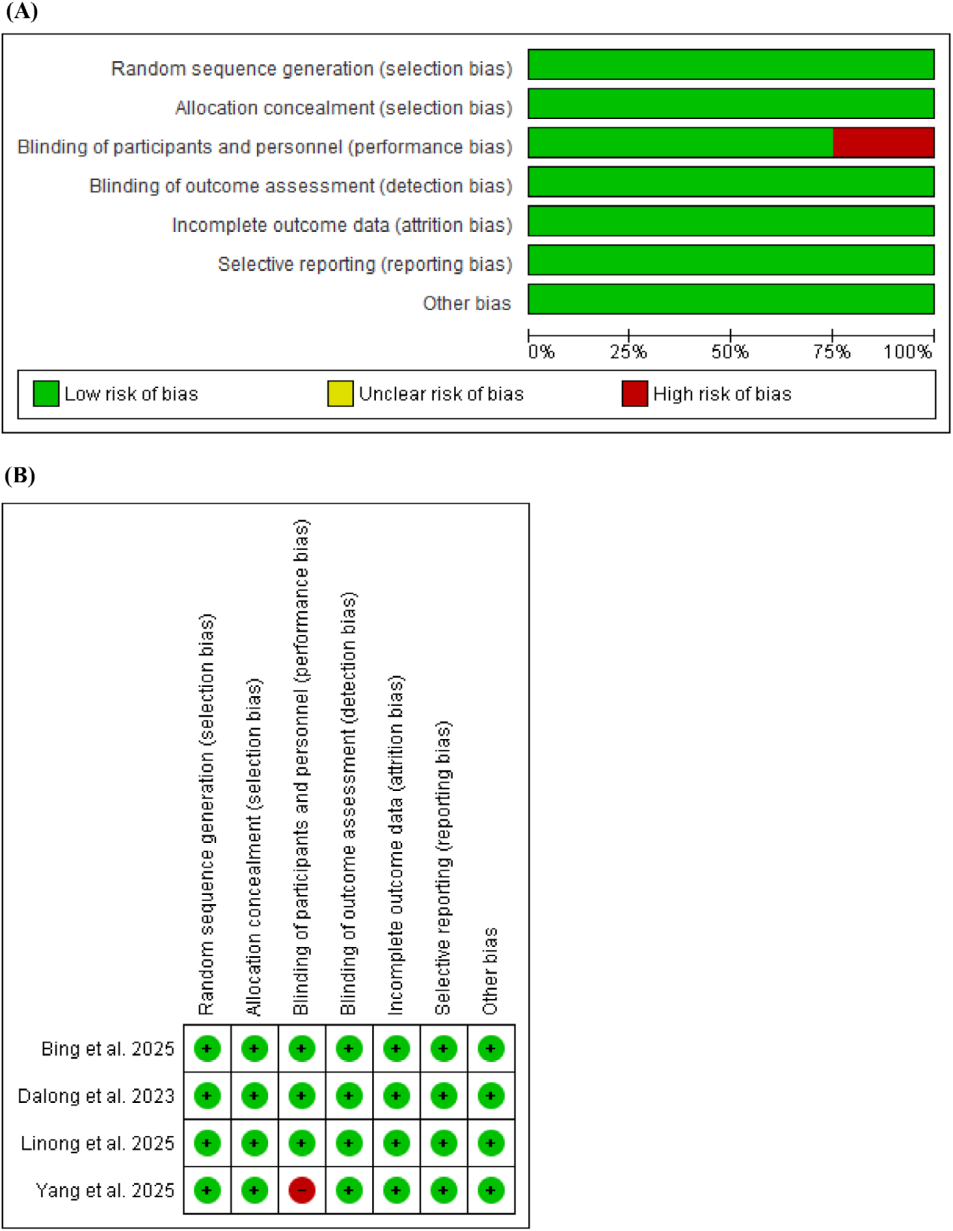
Risk of bias graph (A) and risk of bias summary (B).

The risk of bias was assessed using the Cochrane Risk of Bias Tool. Overall, 3 studies were rated as low risk of bias, whereas 1 study (Yang et al. 2025) [13] was rated as high risk due to its open‐label design. All studies demonstrated adequate randomization using computer‐generated sequences or interactive web response systems with appropriate stratification by baseline HbA1c or BMI. Allocation concealment was ensured through centralized randomization systems in all trials. The main methodological concern was performance bias in Yang et al. 2025, where blinding of participants and personnel was not possible due to differences in injection devices. No concerns were identified regarding incomplete outcome data, selective reporting, or other sources of bias across all included studies. The risk of bias is presented in Figures 2A and 2B and detailed in Table S2. The certainty of evidence for Key outcome was appraised using the GRADE (Grading of Recommendations Assessment, Development and Evaluation) framework (Table S3).

### 3.3 Glycaemic Control Outcomes

**FIGURE 3:**
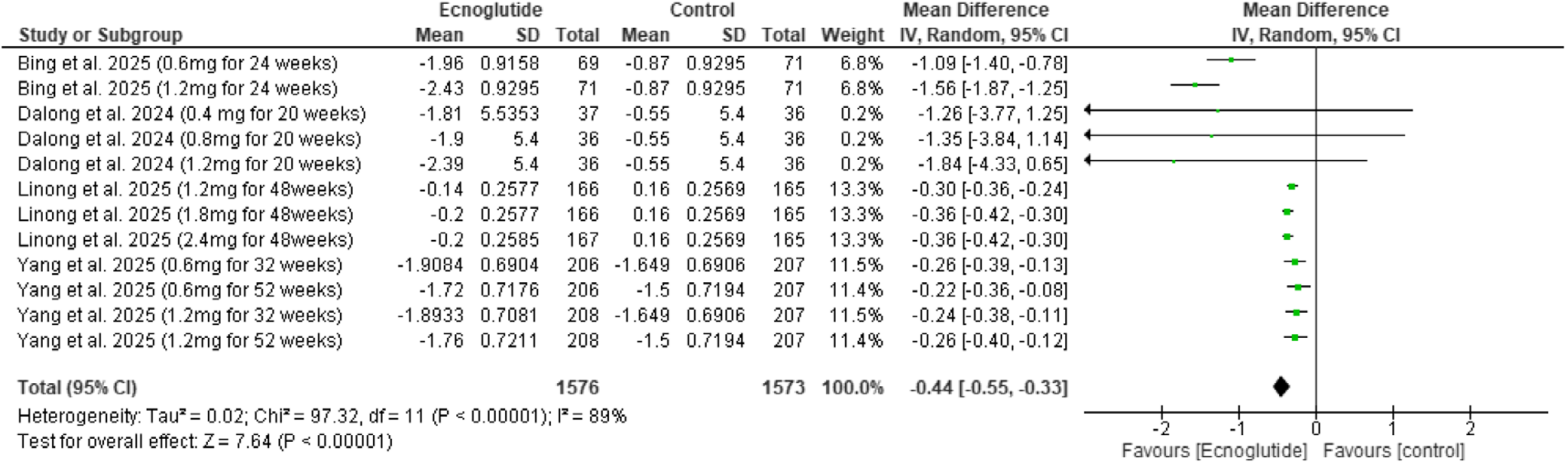
Forest plot of change in HbA1c (%).

**FIGURE 4:**
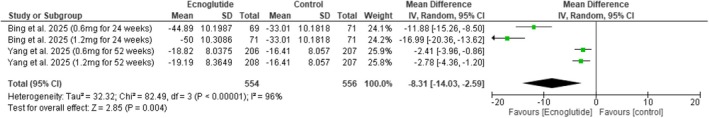
Forest plot of change in HbA1c (mmol/mol).

**FIGURE 5:**
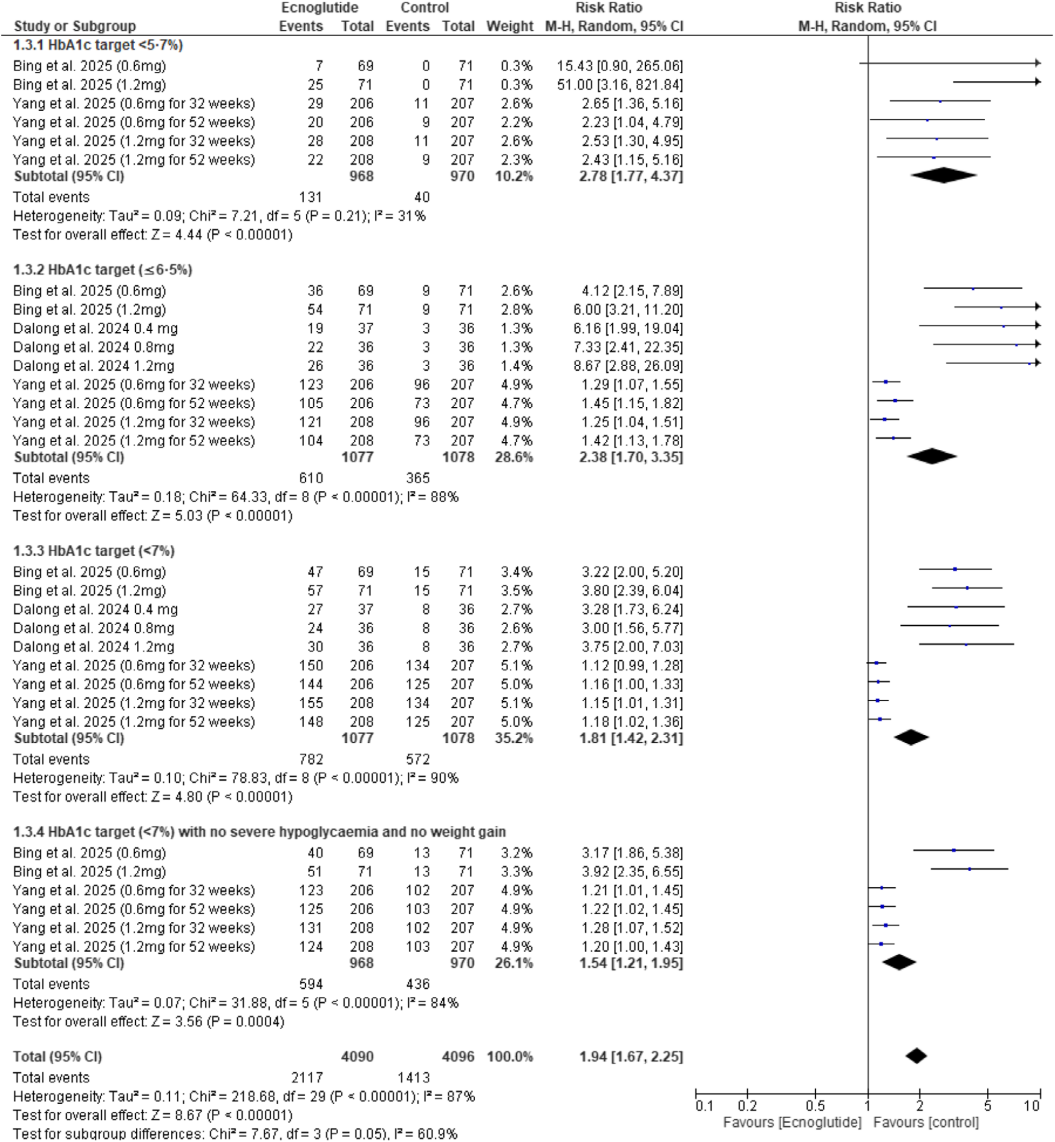
Forest plot of participants achieving HbA1c targets.

Across the included studies, Ecnoglutide was associated with significant reductions in glycaemic control outcomes. HbA1c (%) decreased significantly (MD = −0.44, 95% CI −0.55 to −0.33, *p* < 0.00001) (Figure 3), and HbA1c expressed in mmol/mol showed a similar decline (MD = −8.31, 95% CI −14.03 to −2.59, *p* = 0.004) (Figure 4). The proportion of participants achieving HbA1c targets was lower with Ecnoglutide compared with control (RR = 1.94, 95% CI 1.67 to 2.25, *p* < 0.01) (Figure 5).

**FIGURE 6:**
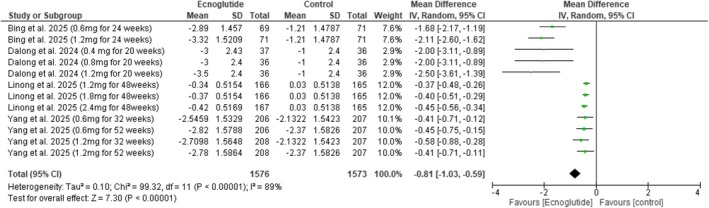
Forest plot of fasting plasma glucose (mmol/L).

Fasting plasma glucose was reduced (MD = −0.81, 95% CI −1.03 to −0.59, *p* < 0.00001) (Figure 6), and 2‐h post‐prandial blood glucose also decreased (MD = −1.98, 95% CI −3.48 to −0.47, *p* = 0.01) (Figure S11). Seven‐point SMBG values showed a significant reduction (MD = −0.91, 95% CI −1.18 to −0.64, *p* < 0.00001) (Figure S12).

### 3.4 Insulin Sensitivity

Improvements were observed in insulin‐related measures. Fasting insulin declined (MD = −3.20, 95% CI −5.47 to −0.93, *p* = 0.006) (Figure S13), and HOMA‐IR was similarly reduced (MD = −1.04, 95% CI −1.62 to −0.46, *p* < 0.01) (Figure S14).

### 3.5 Anthropometric Outcomes

Bodyweight was reduced with Ecnoglutide (MD = −5.63, 95% CI −7.90 to −3.35, *p* < 0.01) (Figure S15), as was percentage change in bodyweight (MD = −7.15, 95% CI −9.98 to −4.32, *p* < 0.01) (Figure S16). A decrease was also observed in BMI (MD = −2.14, 95% CI −3.39 to −0.88, *p* < 0.01) (Figure S17) and waist circumference (MD = −3.84, 95% CI −5.71 to −1.97, *p* < 0.01) (Figure S18).

In contrast, a higher proportion of participants achieved predefined weight‐loss thresholds in the control group (RR = 5.72, 95% CI 4.15 to 7.86, *p* < 0.01) (Figure S19).

### 3.6 Lipid Profile

Significant reductions were observed in percentage change from baseline in total cholesterol (MD = −0.16, 95% CI −0.25 to −0.06, *p* < 0.01) (Figure S110), LDL‐cholesterol (MD = −0.08, 95% CI −0.14 to −0.02, *p* < 0.01) (Figure S111), and triglycerides (MD = −0.37, 95% CI −0.48 to −0.25, *p* < 0.01) (Figure S112). HDL‐cholesterol increased to a greater extent in the control group (MD = 0.05, 95% CI 0.02 to 0.07, *p* < 0.01) (Figure S113).

### 3.7 Liver Enzyme Markers

Reductions were noted in alanine aminotransferase (MD = −9.18, 95% CI −13.12 to −5.23, *p* < 0.01) (Figure S1.14) and aspartate aminotransferase (MD = −3.46, 95% CI −4.97 to −1.95, *p* < 0.01) (Figure S1.15).

### 3.8 Safety Outcomes

Adverse events occurred more frequently in the Ecnoglutide group (RR = 1.09, 95% CI 1.04 to 1.14, *p* < 0.01) (Figure S116). No significant differences were found in serious adverse events (RR = 1.29, 95% CI 0.94 to 1.77, *p* = 0.11) (Figure S117) or treatment‐related serious adverse events (RR = 4.79, 95% CI 0.82 to 27.89, *p* = 0.08) (Figure S118). Adverse events leading to treatment discontinuation were similar between groups (RR = 1.40, 95% CI 0.72 to 2.70, *p* = 0.32) (Figure S119).

### 3.9 Sensitivity Analyses and Heterogeneity

Heterogeneity was assessed for all outcomes, and sensitivity analyses were conducted for those with substantial variability (I^2^ > 50%). Exclusion of influential studies markedly reduced heterogeneity across multiple outcomes. For glycaemic parameters, removing Bing et al., 2025 decreased heterogeneity for HbA1c, fasting plasma glucose, and 2‐h post‐prandial glucose. Insulin‐related outcomes (fasting insulin and HOMA‐IR) became homogeneous after excluding Linong et al., 2025. High heterogeneity in bodyweight‐related measures, BMI, and waist circumference was also substantially reduced following the same exclusion. Similarly, lipid and hepatic biomarkers showed minimal variability after removing influential studies. Overall, these analyses confirmed that the pooled effect estimates were robust, and the direction and significance of effects remained consistent, despite initial heterogeneity (see Figures S2.1–S2.15).

### 3.10 Subgroup Analyses by Dosage

To explore potential dose–response effects, the outcomes were analysed according to Ecnoglutide dosage (0.4 mg, 0.6 mg, 0.8 mg, 1.2 mg, 1.8 mg, 2.4 mg). Ecnoglutide demonstrated strong, dose‐dependent effects on several outcomes. Higher doses consistently produced larger improvements in glycaemic control, with the most pronounced reductions in HbA1c (%) and fasting plasma glucose observed at 1.8 mg and 2.4 mg, whereas lower doses showed smaller effects. Fasting insulin and HOMA‐IR also improved progressively with dose, reflecting enhanced insulin sensitivity at higher doses. Anthropometric measures, including bodyweight, BMI, waist circumference, and percentage bodyweight reduction, exhibited clear dose–response relationships, with maximal reductions at the highest doses. HDL‐C and triglycerides similarly increased or decreased, respectively, in a dose‐dependent manner. Hepatic biomarkers, ALT and AST, showed progressively larger reductions with increasing doses, peaking at 1.8–2.4 mg. These findings indicate that for glycaemic, anthropometric, lipid (HDL‐C and triglycerides), and hepatic outcomes, the effect of Ecnoglutide is strongly modulated by dose.

In contrast, some outcomes were minimally affected by dosage or showed no clear dose–response relationship. HbA1c in mmol/mol and 2‐h post‐prandial glucose demonstrated high variability across doses, with no statistically significant differences between low and high doses. Hip circumference reductions were similar across the lower doses, suggesting limited additional benefit at higher doses. Total cholesterol and LDL‐cholesterol showed modest reductions that were largely independent of dose. These observations indicate that for these specific outcomes, the magnitude of ecnoglutide's effect does not appear to be strongly influenced by dosage, and lower doses provide comparable benefits to higher doses. Detailed results are provided in Figures S3.1–S3.17.

### 3.11 Subgroup Analysis by Follow‐Up Duration

Outcomes were also analysed according to follow‐up duration: short‐term (20–24 weeks), intermediate‐term (32–40 weeks), and long‐term (48–52 weeks). Ecnoglutide's effects on glycaemic, anthropometric, and metabolic outcomes were significantly modulated by follow‐up duration for several measures. HbA1c in mmol/mol, fasting plasma glucose, 2‐h post‐prandial glucose, bodyweight, percentage bodyweight reduction, BMI, and waist circumference all demonstrated clear duration‐dependent effects. Short‐term follow‐up (20–24 weeks) showed substantial initial improvements in glycaemia and bodyweight, which generally increased or were maintained with intermediate‐ and long‐term follow‐up, with the largest effects observed in the long‐term (48–52 weeks) subgroups. Fasting insulin and HOMA‐IR also showed numerically greater reductions with longer follow‐up, though statistical tests for subgroup differences were not significant. These findings indicate that for these outcomes, longer treatment duration enhances the magnitude of benefit.

In contrast, some outcomes were minimally affected by follow‐up duration. HbA1c (%) reductions were significant across all durations but showed no statistically meaningful differences between subgroups. Hip circumference, total cholesterol, LDL‐cholesterol, HDL‐cholesterol, and triglycerides exhibited small or modest changes across follow‐up periods, and tests for subgroup differences were largely non‐significant, indicating that treatment duration did not substantially alter the effect for these outcomes. These results suggest that while Ecnoglutide provides consistent benefits over time for these measures, extending treatment duration does not meaningfully change the magnitude of effect. Detailed results are provided in Figures S4.1–S4.15.

## 4 Discussion

Several novel drugs have been introduced to improve quality of life in individuals with type 2 diabetes mellitus. This study evaluates the efficacy and safety of ecnoglutide, a novel glucagon‐like peptide‐1 receptor agonist (GLP‐1 RA) [21]. Pooled analysis of four randomized controlled trials (RCTs) including 1643 participants demonstrated reductions in HbA1c, body weight, fasting, and postprandial glucose levels compared with placebo. These outcomes suggest that ecnoglutide may be a promising metabolic therapy for achieving glycaemic control and weight reduction, consistent with the effects reported for other GLP‐1 RAs such as semaglutide and dulaglutide. However, the limited number of trials observed heterogeneity; the differences in population characteristics, comparator arms, dosing regimens, and follow‐up durations should be considered when interpreting these findings [22].

Dose stratified analyses indicate apparent trends towards dose‐dependent improvements in outcomes such as HbA1c, body weight, insulin resistance, and hepatic biomarkers, reflecting biological plausibility and enhanced receptor engagement at higher doses [23]. Nevertheless, these subgroup analyses were exploratory, and a formal dose response meta‐analysis was not feasible due to the small number of trials.

Ecnoglutide participants experienced reductions in fasting blood glucose and HbA1c levels, with a higher proportion achieving HbA1c < 7% compared with control groups. While these findings support the glycaemic efficacy of ecnoglutide in treatment‐naïve or metformin‐treated individuals, comparisons with high‐dose semaglutide reported in SUSTAIN trials or other GLP‐1 RAs should be interpreted cautiously, as no direct head‐to‐head trials or network meta‐analyses were conducted [24, 25]. Similarly, improvements in fasting insulin and HOMA‐IR levels suggest enhanced insulin sensitivity, though indirect comparisons with other GLP‐1 RAs remain exploratory.

Participants also experienced weight reductions alongside decreases in waist circumference and BMI [26, 27]. While these effects appear consistent with the known GLP‐1 receptor‐mediated mechanisms, comparisons of magnitude with other agents (e.g., semaglutide in STEP trials) are indirect and intended for contextual discussion rather than evidence of superiority [28]. One study reported a higher proportion of weight‐loss responders in the control group, likely reflecting study specific factors or reporting variability. These observations highlight the need for future studies with weight‐specific endpoints to confirm and quantify weight‐reducing effects.

Ecnoglutide also showed favourable trends in lipid metabolism, with reductions in total cholesterol, LDL‐C, and triglycerides, and modest improvements in HDL‐C [12]. Hepatic biomarkers such as ALT and AST declined in line with patterns observed with other GLP‐1 RAs, supporting potential benefits in metabolic‐associated steatotic liver disease (MASLD) [29]. These findings are consistent with known pharmacologic effects, though no direct comparative evidence is available.

The safety profile of ecnoglutide was generally consistent with other GLP‐1 RAs. Mild to moderate gastrointestinal adverse events, including nausea, diarrhoea, and reduced appetite, were observed, while serious adverse events such as pancreatitis, major hypoglycaemia, or thyroid‐related events were not reported. These observations suggest a tolerable safety profile at the doses studied [30]. However, the limited number of trials and inconsistent reporting of dose‐specific adverse events preclude definitive conclusions regarding rare or long‐term safety outcomes.

Overall, these findings suggest that ecnoglutide may be a promising incretin‐based therapy with potential metabolic benefits. Dose‐dependent trends and improvements in glycaemic and anthropometric measures support further investigation. Importantly, comparisons with other GLP‐1 RAs should be interpreted as **indirect and exploratory**, given the absence of head‐to‐head trials or cardiovascular outcome data. Future studies are needed to evaluate cardiovascular and renal outcomes, long‐term safety, and real‐world adherence to fully establish the clinical profile of ecnoglutide.

## 5 Limitations

This meta‐analysis has several important limitations that should be acknowledged. First, only four randomized controlled trials comprising 1643 participants were included. This limited evidence base substantially restricts statistical power, limits the precision of pooled estimates, and prevents reliable assessment of rare or infrequent adverse events [31]. With a small number of trials, pooled estimates remain susceptible to evidential fragility, small‐study effects, and potential overestimation of treatment effects. In the primary analysis, different ecnoglutide doses were pooled to preserve statistical power, as the limited number of trials and uneven dose distribution precluded reliable dose‐specific pooled estimates. Although dose‐stratified subgroup analyses were performed, pooling across doses may have obscured potential dose‐dependent differences and should be interpreted with caution. Second, clinical heterogeneity represents a central limitation of this analysis. Despite moderate statistical heterogeneity in several pooled analyses, there was considerable variability across trials in baseline patient characteristics, disease duration, background glucose‐lowering therapies, and study duration [32]. Such variability may confound pooled efficacy and safety estimates and limit direct comparability across studies.

Third, the included populations were relatively homogeneous, with most trials enrolling individuals with shorter diabetes duration and excluding patients with advanced renal impairment or significant cardiovascular comorbidities. This restricts the external validity and generalizability of our findings to broader, higher‐risk real‐world populations.

Fourth, one included phase 3 trial used an open‐label design, introducing unavoidable performance and detection bias [33]. Lack of blinding may particularly influence subjective outcomes such as weight‐related behaviours, dietary adherence, and patient‐reported adverse events, especially gastrointestinal symptoms. Participants aware of receiving active treatment may modify lifestyle behaviours or report adverse events differently, potentially exaggerating treatment effects or safety signals. Although sensitivity analyses did not materially alter pooled estimates, the influence of open‐label design remains a significant methodological limitation and should be considered when interpreting the findings.

Fifth, most included studies had relatively short follow‐up durations (< 40 weeks), limiting evaluation of long‐term cardiovascular safety, durability of glycaemic control, and sustained weight effects [34]. Longer outcome‐driven trials are required to establish the long‐term benefit–risk profile of ecnoglutide.

Sixth, the higher incidence of gastrointestinal adverse events observed with ecnoglutide, consistent with the known class effects of GLP‐1 receptor agonists, may influence treatment adherence and real‐world tolerability [35]. However, long‐term adherence data remain limited.

Finally, the absence of a formal dose–response meta‐analysis due to limited and inconsistently reported dose regimens across trials precludes quantitative assessment of dose‐dependent efficacy and safety endpoints. This further underscores the need for cautious interpretation of pooled findings.

Taking together, although current evidence suggests promising metabolic benefits, the limited number of trials and relatively small cumulative sample size highlight the need for larger, well‐powered, long‐term randomized controlled trials to confirm these findings.

## 6 Conclusion

This meta‐analysis highlights the potential clinical benefits of ecnoglutide, a novel GLP‐1 analogue, in adults with type 2 diabetes mellitus. The available evidence suggests improvements in glycaemic control, body weight, lipid parameters, and hepatic biomarkers. The safety profile appears generally consistent with other GLP‐1 receptor agonists, with mostly mild to moderate gastrointestinal adverse events reported. Comparisons with other GLP‐1 analogues should be interpreted cautiously, as no direct head‐to‐head trials or cardiovascular outcome data are currently available. Although these findings are promising, they are based on a limited number of trials and relatively short follow‐up durations. Future research, including long‐term cardiovascular outcome studies and well‐powered head‐to‐head comparisons, is needed to more fully evaluate the therapeutic potential, safety, and real‐world applicability of ecnoglutide.

## Author Contributions

**Mukesh Kumar:** Conceptualization, Methodology, Data curation, Formal analysis, Writing – original draft, Software. **Sumet Kumar:** Conceptualization, Methodology, Software, Writing – original draft, Visualization. **Sheikh Muhammad Ebtehaj Ali:** Writing – original draft, Data Curation, Formal analysis, Software. **Arshia Arif Janjua:** writing – original draft, formal analysis, software. **Hadia Qazi:** writing – original draft, formal analysis, software. **Fatima Hanif:** methodology, writing – original draft. **Sana Mohammad Ali:** writing – original draft, methodology. **Rayan Babar:** writing – original draft, visualization, data curation. **Laksh Kumar:** writing – review and editing. **Rumaisa Imtiaz Sheikh:** methodology, writing – original draft. **Hanzlah Ahmed:** writing – original draft, visualization, data curation. **Aayush Chaulagain:** supervision, writing – review and editing. **Khizra Gul:** writing – original draft. **Ayesha Sarwar Ali:** Writing – original draft. **Shehzadi Umme Rubab Bukhari:** writing – original draft. **Adarsh Raja:** supervision, writing – review and editing, validation.

## Funding

The authors have nothing to report.

## Consent

The authors have nothing to report.

## Conflicts of Interest

The authors declare no conflicts of interest.

## Supporting information


**Table S1:** Search strategy.
**Table S2:** Risk of bias assessment.
**Table S3:** GRADE table for outcomes.
**Figure S1:** 1: 2‐h post‐prandial blood glucose, mmol/L.
**Figure S1:** 2: 7‐point SMBG, mmol/L.
**Figure S1:** 3: Fasting insulin, μU/mL.
**Figure S1:** 4: HOMA‐IR.
**Figure S1:** 5: Absolute change in Bodyweight, kg.
**Figure S1:** 6: Percentage change in bodyweight.
**Figure S1:** 7: BMI, kg/m2.
**Figure S1:** 8: Waist circumference, cm.
**Figure S1:** 9: Participants with % bodyweight reduction.
**Figure S1:** 10: Percentage change from baseline in total cholesterol.
**Figure S1:** 11: Percentage change from baseline in LDL‐cholesterol.
**Figure S1:** 12: Triglycerides, mmol/L.
**Figure S1:** 13: Percentage change from baseline in HDL‐cholesterol.
**Figure S1:** 14: Alanine aminotransferase, U/L.
**Figure S1:** 15: Aspartate aminotransferase, U/L.
**Figure S1:** 16: Any adverse events.
**Figure S1:** 17: Serious adverse events.
**Figure S1:** 18: Treatment‐related serious adverse events.
**Figure S1:** 19: Adverse events leading to treatment discontinuation.
**Figure S2:** 1: HbA1C % (‐Bing et al. 2025).
**Figure S2:** 2: HbA1c, mmol/mol (‐Bing et al. 2025).
**Figure S2:** 3: Fasting plasma glucose, mmol/L (‐Bing et al. 2025).
**Figure S2:** 4: 2 h post‐prandial blood glucose, mmol/L (‐Bing et al. 2025).
**Figure S2:** 5: fasting insulin, μU/mL (‐Linong et al. 2025).
**Figure S2:** 6: HOMA‐IR (‐Linong et al. 2025).
**Figure S2:** 7: Bodyweight, kg (‐Linong et al. 2025).
**Figure S2:** 8: Percentage changes in bodyweight (‐Linong et al. 2025).
**Figure S2:** 9: BMI, kg/m2 (‐Linong et al. 2025).
**Figure S2:** 10: Waist circumference, cm (‐Linong et al. 2025).
**Figure S2:** 11: Percentage change from baseline in total cholesterol (‐Linong et al. 2025).
**Figure S2:** 12: Percentage change from baseline in HDL‐cholesterol (‐Linong et al. 2025).
**Figure S2:** 13: Triglycerides, mmol/L (‐Linong et al. 2025).
**Figure S2:** 14: Alanine aminotransferase, U/L (‐Linong et al. 2025).
**Figure S2:** 15: Aspartate aminotransferase, U/L (‐Linong et al. 2025) Forest Plot of Subgroup Analysis by Dosage.
**Figure S3:** 1: HbA1C %.
**Figure S3:** 2: HbA1c, mmol/mol.
**Figure S3:** 3: Fasting plasma glucose, mmol/L.
**Figure S3:** 4: 2 h post‐prandial blood glucose, mmol/L.
**Figure S3:** 5: Fasting insulin, μU/mL.
**Figure S3:** 6: HOMA‐IR.
**Figure S3:** 7: Bodyweight, kg.
**Figure S3:** 8: Percentage change in bodyweight.
**Figure S3:** 9: BMI, kg/m2.
**Figure S3:** 10: Waist circumference, cm.
**Figure S3:** 11: Hip circumference, cm.
**Figure S3:** 12: Percentage change from baseline in total cholesterol.
**Figure S3:** 13: Percentage change from baseline in LDL‐cholesterol.
**Figure S3:** 14: Percentage change from baseline in HDL‐cholesterol.
**Figure S3:** 15: Triglycerides, mmol/L.
**Figure S3:** 16: Alanine aminotransferase, U/L.
**Figure S3:** 17: Aspartate aminotransferase, U/L Forest Plot of Subgroup Analysis by Follow‐up Duration.
**Figure S4:** 1: HbA1C %.
**Figure S4:** 2: HbA1c, mmol/mol.
**Figure S4:** 3: Fasting plasma glucose, mmol/L.
**Figure S4:** 4: 2 h post‐prandial blood glucose, mmol/L.
**Figure S4:** 5: Fasting insulin, μU/mL.
**Figure S4:** 6: HOMA‐IR.
**Figure S4:** 7: Bodyweight, kg.
**Figure S4:** 8: Percentage change in bodyweight.
**Figure S4:** 9: BMI, kg/m2 10: Waist circumference, cm.
**Figure S4:** 11: Hip circumference, cm.
**Figure S4:** 12: Percentage change from baseline in total cholesterol.
**Figure S4:** 13: Percentage change from baseline in LDL‐cholesterol.
**Figure S4:** 14: Percentage change from baseline in HDL‐cholesterol.
**Figure S4:** 15: Triglycerides, mmol/L.

## Data Availability

The data that supports the findings of this study are available in the Supporting Information of this article.
