# Supplementary material for: Comparative Efficacy and Safety of Ecnoglutide in Type 2 Diabetes: A Systematic Review and Meta‐Analysis
Source: Endocrinol Diabetes Metab. 2026 Apr 5;9(3):e70217. doi: 10.1002/edm2.70217 (PMC13052322; doi:10.1002/edm2.70217)

**Supplementary Material**

**Supplementary Table 1: Search Strategy**

| PubMed (n=7) | "ecnoglutide"[All Fields] AND ("diabete"[All Fields] OR "diabetes mellitus"[MeSH Terms] OR ("diabetes"[All Fields] AND "mellitus"[All Fields]) OR "diabetes mellitus"[All Fields] OR "diabetes"[All Fields] OR "diabetes insipidus"[MeSH Terms] OR ("diabetes"[All Fields] AND "insipidus"[All Fields]) OR "diabetes insipidus"[All Fields] OR "diabetic"[All Fields] OR "diabetics"[All Fields] OR "diabets"[All Fields]) |
| --- | --- |
| Cochrane Library (n=5) | (ecnoglutide) AND (diabetes) |
| Google Scholar (n=112) | (ecnoglutide) AND (diabetes) |
| Science Direct (n=14) | (ecnoglutide) AND (diabetes) |
| Clinicaltrials.gov (n=3) | (ecnoglutide) AND (diabetes) |

**Supplementary Table 2: Risk of Bias Assessment**

|  | Cochrane Risk-of-Bias | | |
| --- | --- | --- | --- |
|  | Bias | Risk of Bias | Author Judgement |
| Linong et al. 2025 | Random sequence generation (selection bias) | Low Risk | Participants were randomly assigned (3:3:3:1:1:1) to ecnoglutide or volume-matched placebo using a computer-generated interactive web response system with BMI stratification. |
|  | Allocation concealment (selection bias) | Low Risk | Centralised randomisation via web-based system; double-blind placebo-controlled design ensured concealment. |
|  | Blinding of participants and personnel (performance bias) | Low risk | Participants, investigators, and sponsors were masked throughout treatment; placebo identical in appearance; dose modifications protocolised; intention-to-treat analysis used; no evidence of unblinding or differential co-interventions. |
|  | Blinding of outcome assessment (detection bias) | Low Risk | Objective outcomes (body weight, labs) measured at clinic visits; study personnel masked; outcomes largely objective |
|  | Incomplete outcome data (attrition bias) | Low Risk | No incomplete outcome data was reported, minimizing the risk of attrition bias. |
|  | Selective reporting (reporting bias) | Low Risk | There is no evidence of selective outcome reporting, reducing the risk of reporting bias. |
|  | Other bias | Low Risk | No other biases were identified in the study that could significantly impact the results. |
| Dalong et al. 2023 | Random sequence generation (selection bias) | Low Risk | Participants were randomly assigned (1:1:1:1) to 0.4, 0.8, or 1.2 mg ecnoglutide or placebo using a SAS-generated randomization list stratified by baseline HbA1c, via a centralized interactive web response system. |
|  | Allocation concealment (selection bias) | Low Risk | Centralized randomization and dispensing through the web response system; study drugs and placebo were identically labeled and indistinguishable, ensuring concealment. |
|  | Blinding of participants and personnel (performance bias) | Low Risk | Participants, investigators, study staff, and sponsor were masked to treatment; placebo matched for volume and appearance; dose escalation protocolized; analyses intention-to-treat. No evidence of unblinding or differential co-interventions. |
|  | Blinding of outcome assessment (detection bias) | Low risk | Outcomes (HbA1c, glucose, weight, lipids) measured objectively at study visits; personnel masked to allocation |
|  | Incomplete outcome data (attrition bias) | Low Risk | No incomplete outcome data was reported, minimizing the risk of attrition bias. |
|  | Selective reporting (reporting bias) | Low Risk | There is no evidence of selective outcome reporting, reducing the risk of reporting bias. |
|  | Other bias | Low Risk | No other biases were identified in the study that could significantly impact the results. |
| Bing et al. 2025 | Random sequence generation (selection bias) | Low Risk | Randomisation (2:2:1:1 ratio) was done via an interactive web response system with stratification by baseline HbA1c |
|  | Allocation concealment (selection bias) | \| Low risk \| \| --- \|  \|  \| \| --- \| | Centralised IWRS ensured allocation concealment. Investigators and participants could not predict assignments |
|  | Blinding of participants and personnel (performance bias) | Low Risk | Double-blind design; active drug and placebo provided in identical injector pens; investigators, participants, and sponsor blinded to allocation |
|  | Blinding of outcome assessment (detection bias) | Low Risk | Primary and secondary endpoints (HbA1c, glucose, weight, etc.) assessed by central laboratory and blinded adjudication |
|  | Incomplete outcome data (attrition bias) | Low Risk | No incomplete outcome data was reported, minimizing the risk of attrition bias. |
|  | Selective reporting (reporting bias) | Low Risk | There is no evidence of selective outcome reporting, reducing the risk of reporting bias. |
|  | Other bias | Low Risk | No other biases were identified in the study that could significantly impact the results. |
| Yang et al. 2025 | Random sequence generation (selection bias) | Low Risk | Participants were randomized 1:1:1 using an independent statistician and an interactive web response system |
|  | Allocation concealment (selection bias) | \| Low risk \| \| --- \|  \|  \| \| --- \| | Centralized randomization system (IWRS) was used. Investigators did not control assignment. |
|  | Blinding of participants and personnel (performance bias) | High Risk | The trial was **open label** due to differences in injection devices |
|  | Blinding of outcome assessment (detection bias) | Low Risk | HbA1c and key endpoints were measured centrally and blinded to study sites/sponsors, minimizing detection bias |
|  | Incomplete outcome data (attrition bias) | Low Risk | No incomplete outcome data was reported, minimizing the risk of attrition bias. |
|  | Selective reporting (reporting bias) | Low Risk | There is no evidence of selective outcome reporting, reducing the risk of reporting bias. |
|  | Other bias | Low Risk | No other biases were identified in the study that could significantly impact the results. |

**Supplementary Table 3: GRADE Table for Outcomes**

| **Outcome** | **No. of studies (participants)** | **Relative effect** | **95% CI** | **Absolute effect** | **Certainty of evidence (GRADE)** | **Comments** |
| --- | --- | --- | --- | --- | --- | --- |
| HbA1c (%), change from baseline | 4 RCTs (n = 1,643) | MD −0.44 | −0.55 to −0.33 | — | ⬤⬤⬤⬤ **High** | Consistent effect; narrow CI; robustness confirmed by sensitivity analyses |
| Fasting plasma glucose | 4 RCTs (n = 1,643) | MD −0.81 | −1.03 to −0.59 | — | ⬤⬤⬤⬤ **High** | Strong statistical significance; heterogeneity resolved in sensitivity analysis |
| Bodyweight change | 4 RCTs (n = 1,643) | MD −5.63 | −7.90 to −3.35 | — | ⬤⬤⬤◯ **Moderate** | Initial heterogeneity; downgraded one level for inconsistency |
| Percentage bodyweight change | 3 RCTs (n = 1,538) | MD −7.15 | −9.98 to −4.32 | — | ⬤⬤⬤◯ **Moderate** | Dose-response present but heterogeneity across trials |
| BMI change | 2 RCTs (n = 1287) | MD −2.14 | −3.39 to −0.88 | — | ⬤⬤⬤◯ **Moderate** | Inconsistency reduced after exclusion of influential study |
| Any adverse event | 4 RCTs (n = 1162) | RR 1.09 | 1.04 to 1.14 | 90 more per 1,000 | ⬤⬤⬤⬤ **High** | Precise estimate; consistent increase; no serious bias concerns |
| Serious adverse events | 4 RCTs (n = 1089) | RR 1.29 | 0.94 to 1.77 | — | ⬤⬤◯◯ **Low** | Downgraded for imprecision (CI crosses null) |

**Forest Plot of Secondary Outcomes**

**Supplementary Figure 1.1: 2-hour post-prandial blood glucose, mmol/L**
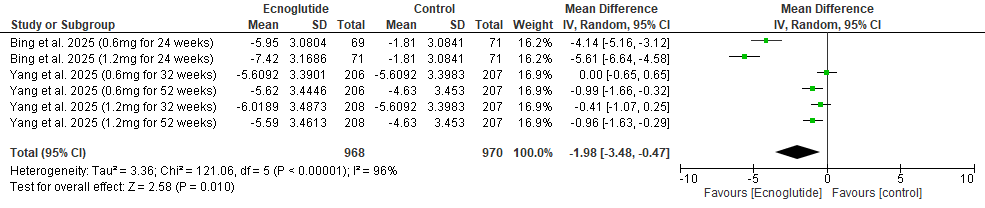


**Supplementary Figure 1.2: 7-point SMBG, mmol/L**
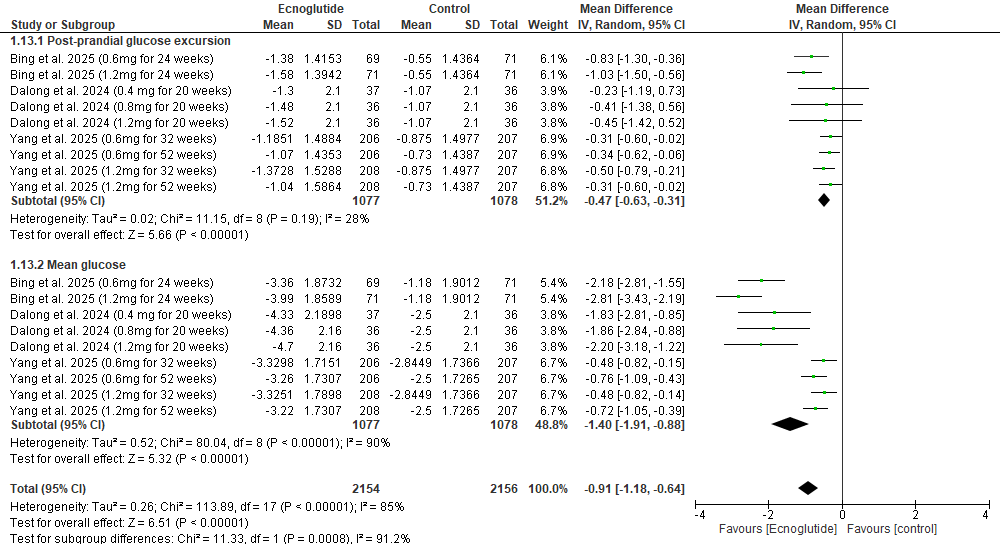


**Supplementary Figure 1.3: Fasting insulin, μU/mL**
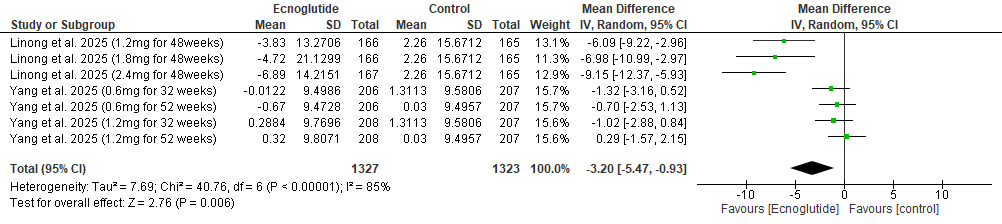


**Supplementary Figure 1.4: HOMA-IR**
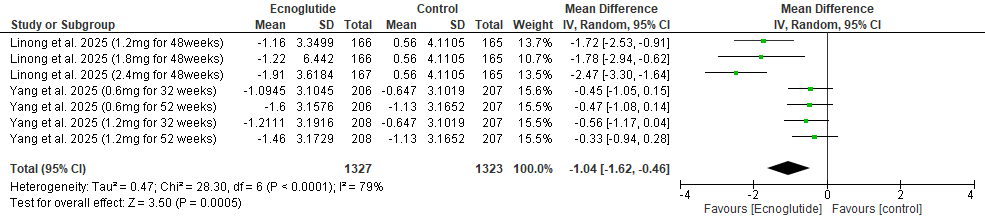


**Supplementary Figure 1.5: Absolute change in Bodyweight, kg**
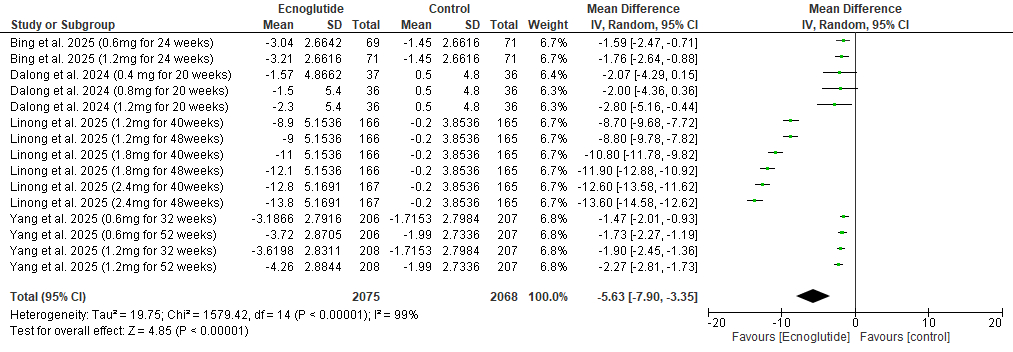


**Supplementary Figure 1.6: Percentage change in bodyweight**
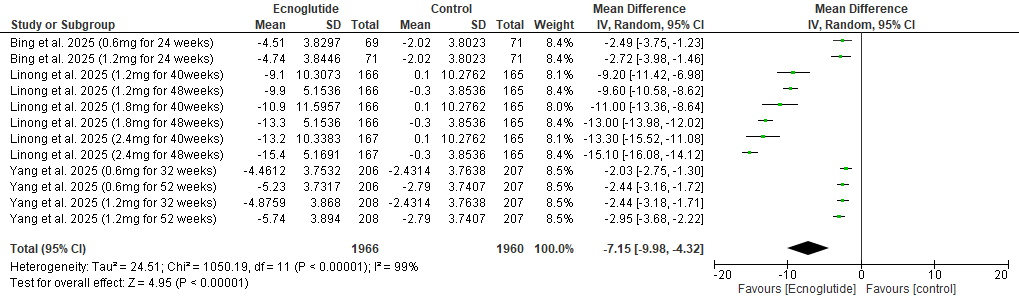


**Supplementary Figure 1.7: BMI, kg/m²**
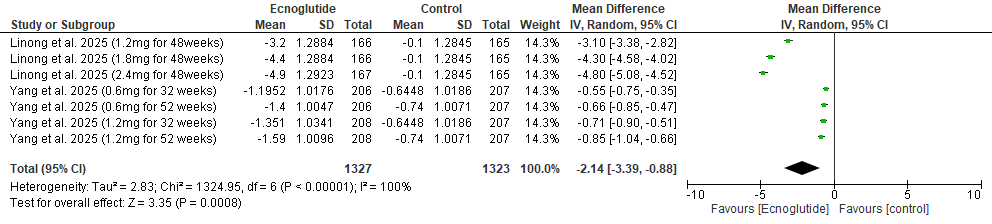


**Supplementary Figure 1.8: Waist circumference, cm**
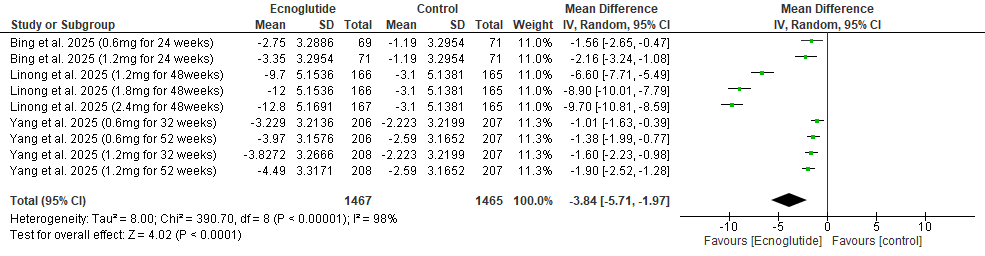


**Supplementary Figure 1.9: Participants with % bodyweight reduction**
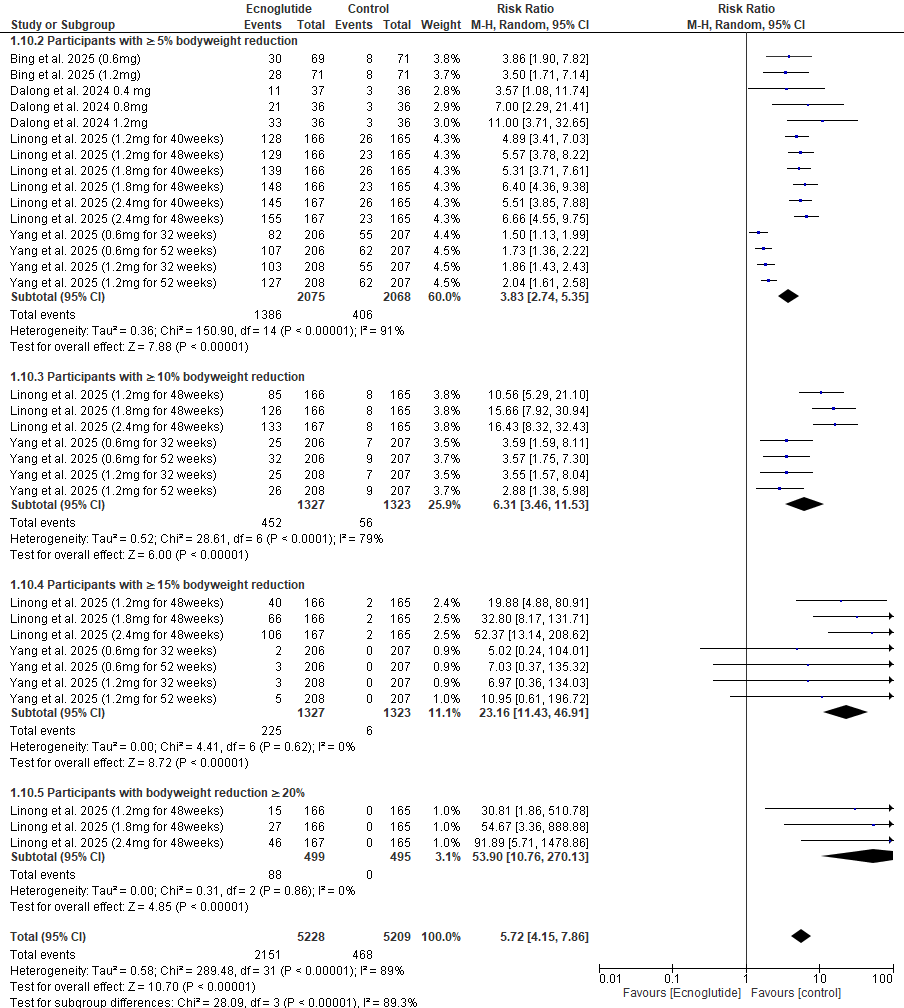


**Supplementary Figure 1.10: Percentage change from baseline in total cholesterol**
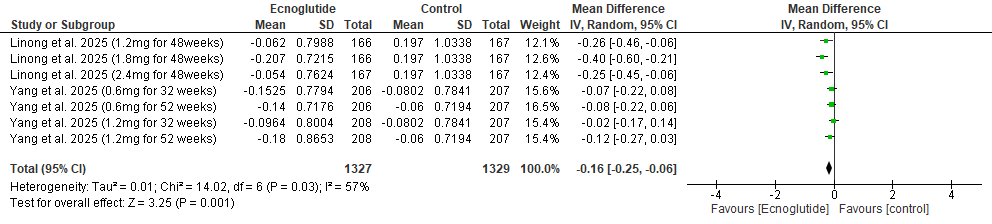


**Supplementary Figure 1.11: Percentage change from baseline in LDL-cholesterol**
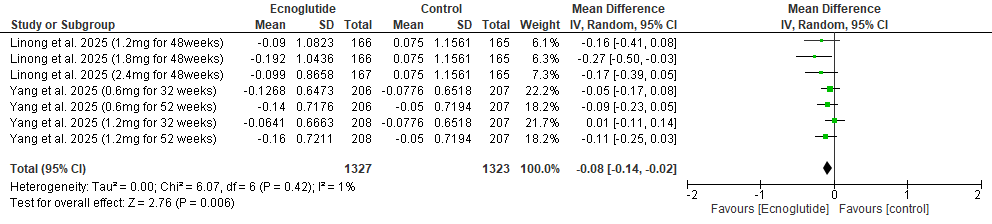


**Supplementary Figure 1.12: Triglycerides, mmol/L**
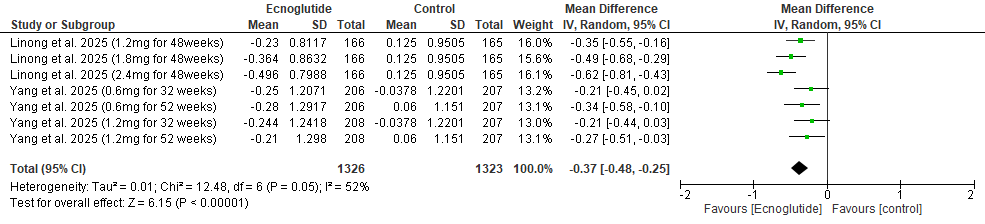


**Supplementary Figure 1.13: Percentage change from baseline in HDL-cholesterol**
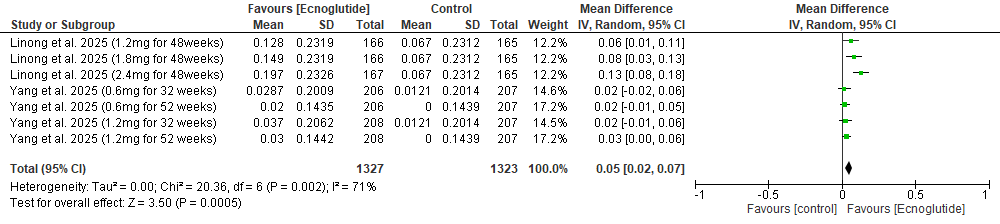


**Supplementary Figure 1.14: Alanine aminotransferase, U/L**
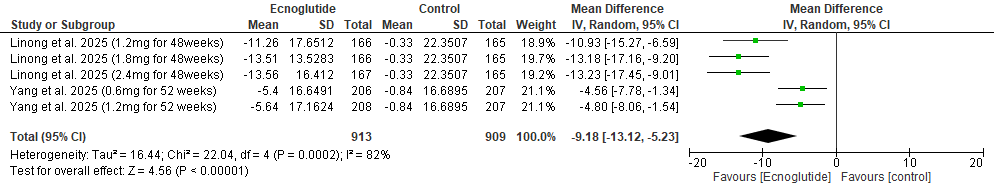


**Supplementary Figure 1.15: Aspartate aminotransferase, U/L**
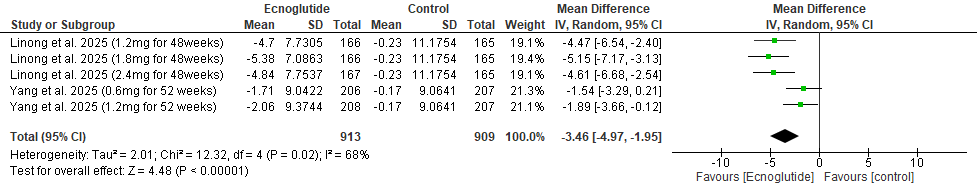


**Supplementary Figure 1.16: Any adverse events**
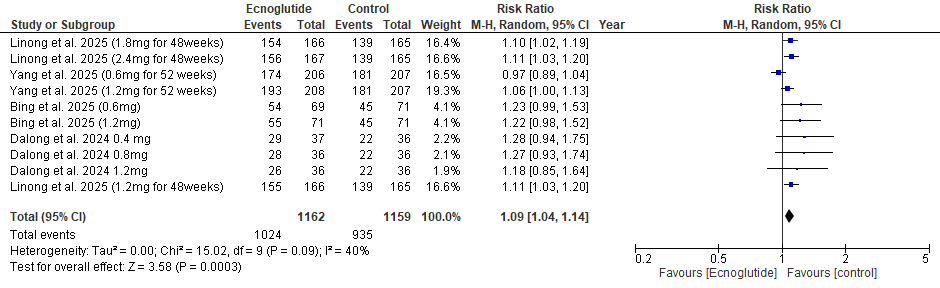


**Supplementary Figure 1.17: Serious adverse events**
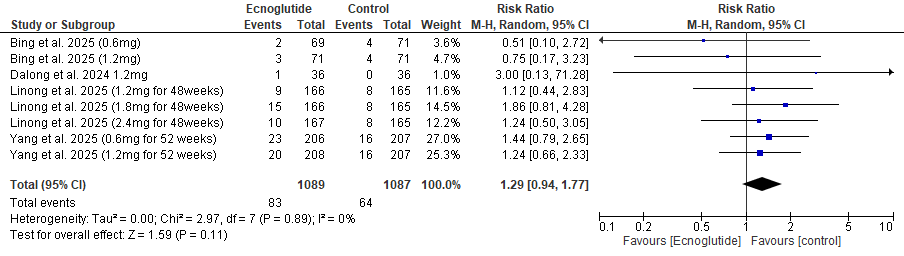


**Supplementary Figure 1.18: Treatment-related serious adverse events**
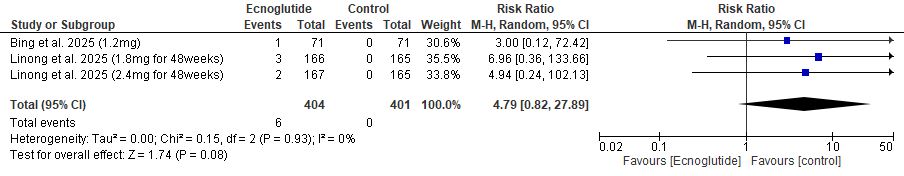


**Supplementary Figure 1.19: Adverse events leading to treatment discontinuation**
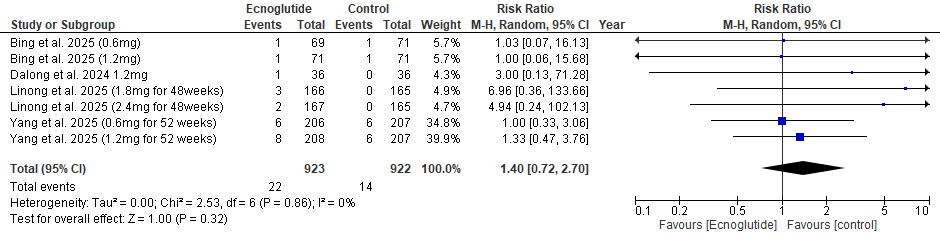


**Forest Plot of Sensitivity Analysis**

**Supplementary Figure 2.1: HbA1C % (-Bing et al. 2025)
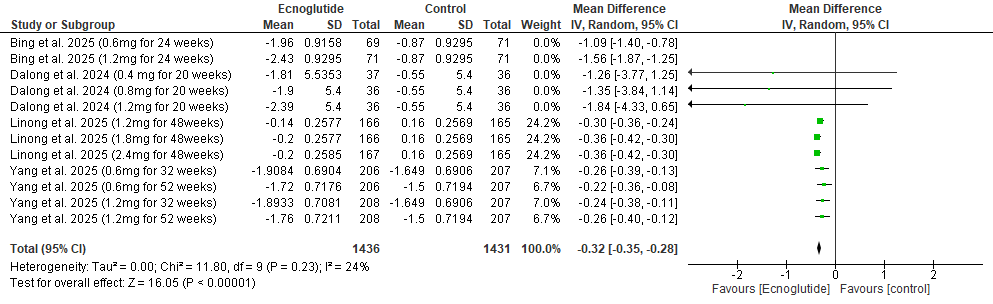
**

**Supplementary Figure 2.2: HbA1c, mmol/mol (-Bing et al. 2025)
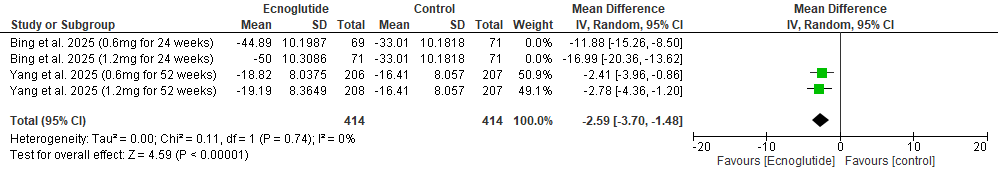
**

**Supplementary Figure 2.3: Fasting plasma glucose, mmol/L (-Bing et al. 2025)
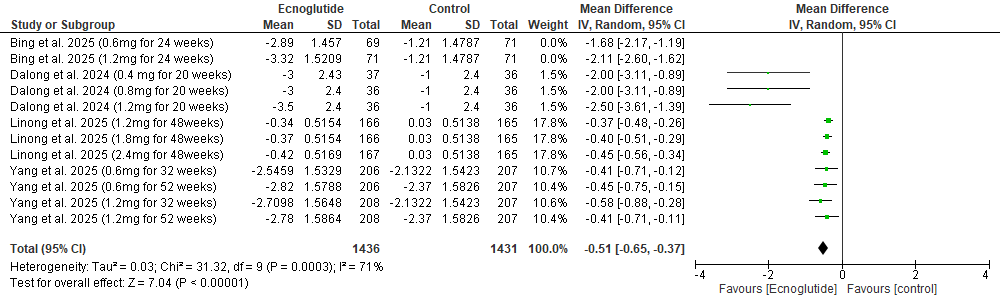
**

**Supplementary Figure 2.4: 2 h post-prandial blood glucose, mmol/L (-Bing et al. 2025)**
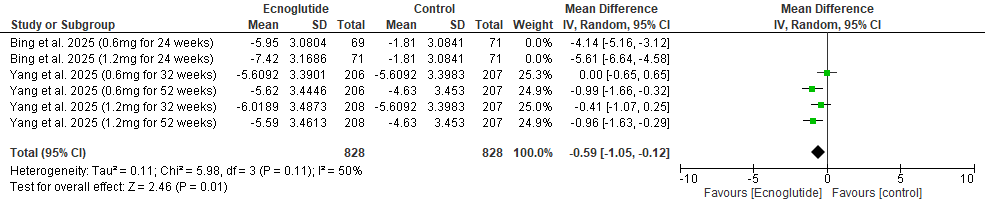


**Supplementary Figure 2.5: fasting insulin, μU/mL (-Linong et al. 2025)**
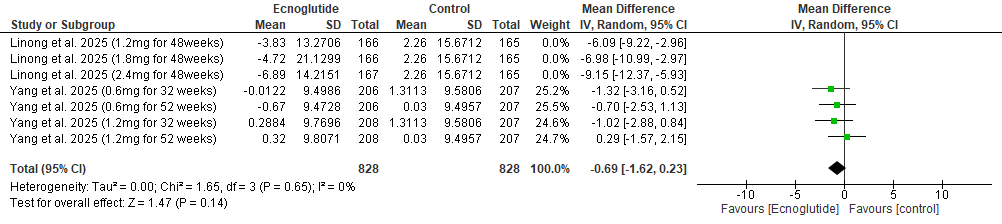


**Supplementary Figure 2.6: HOMA-IR (-Linong et al. 2025)**
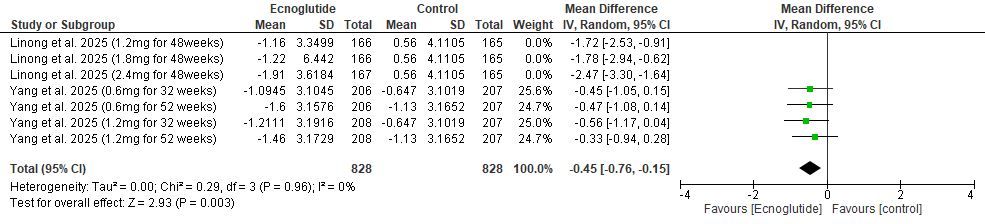


**Supplementary Figure 2.7: Bodyweight, kg (-Linong et al. 2025)
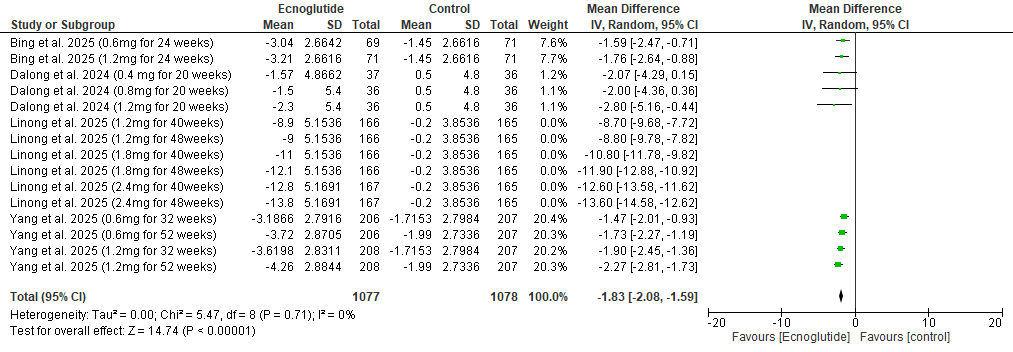
**

**Supplementary Figure 2.8: Percentage changes in bodyweight (-Linong et al. 2025)**
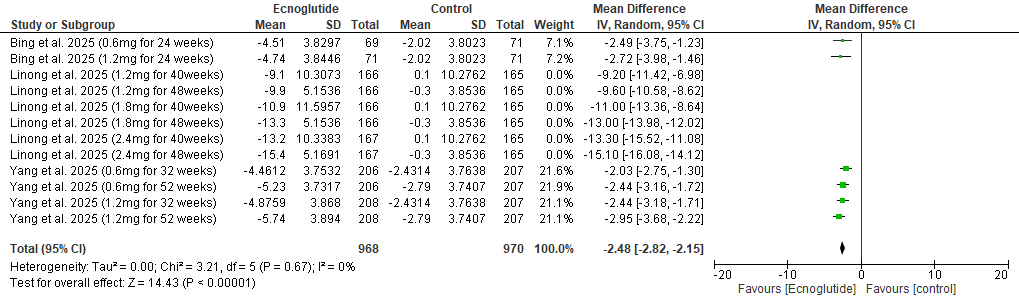


**Supplementary Figure 2.9: BMI, kg/m² (-Linong et al. 2025)
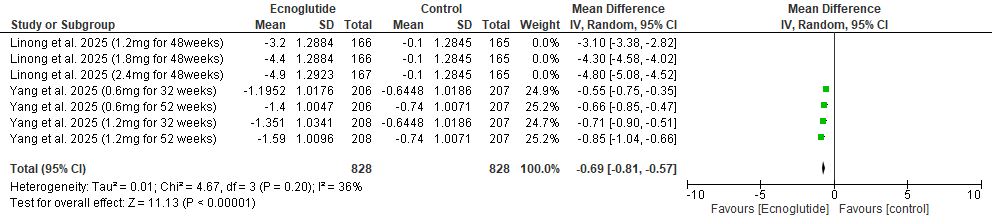
**

**Supplementary Figure 2.10: Waist circumference, cm (-Linong et al. 2025)
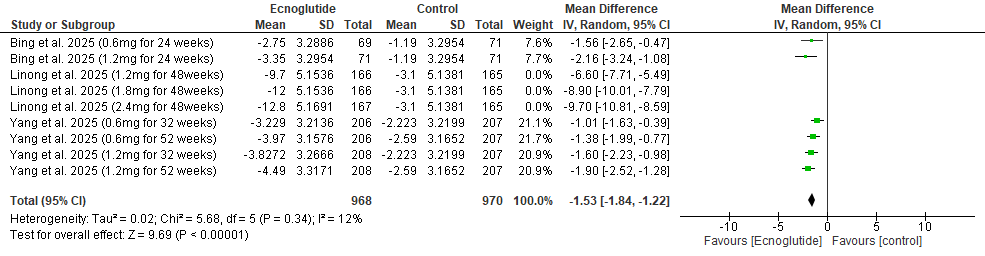
**

**Supplementary Figure 2.11: Percentage change from baseline in total cholesterol (-Linong et al. 2025)
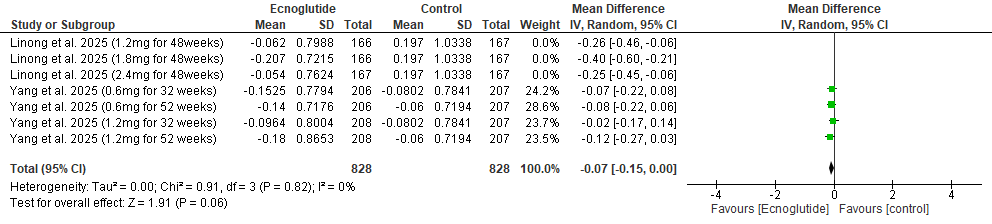
**

**Supplementary Figure 2.12: Percentage change from baseline in HDL-cholesterol (-Linong et al. 2025)
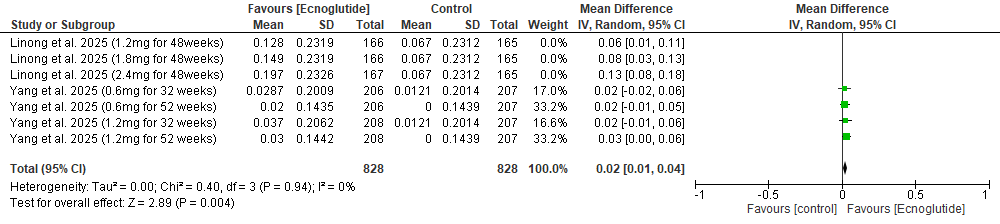
**

**Supplementary Figure 2.13: Triglycerides, mmol/L (-Linong et al. 2025)**
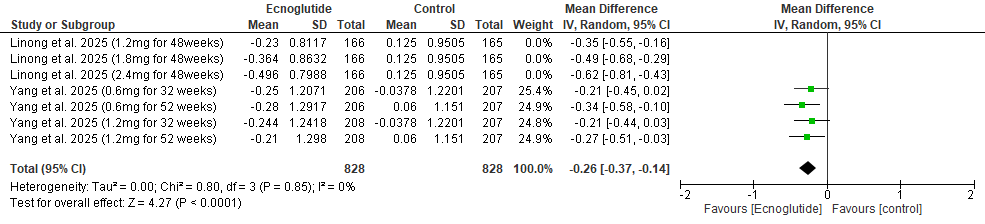


**Supplementary Figure 2.14: Alanine aminotransferase, U/L (-Linong et al. 2025)**
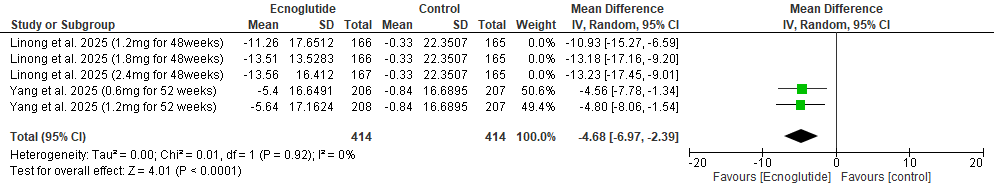


**Supplementary Figure 2.15: Aspartate aminotransferase, U/L (-Linong et al. 2025)**
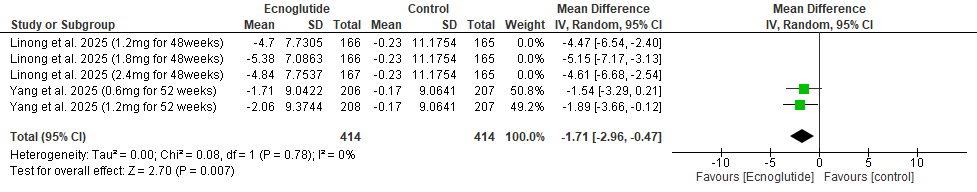


**Forest Plot of Subgroup Analysis by Dosage**

**Supplementary Figure 3.1: HbA1C %
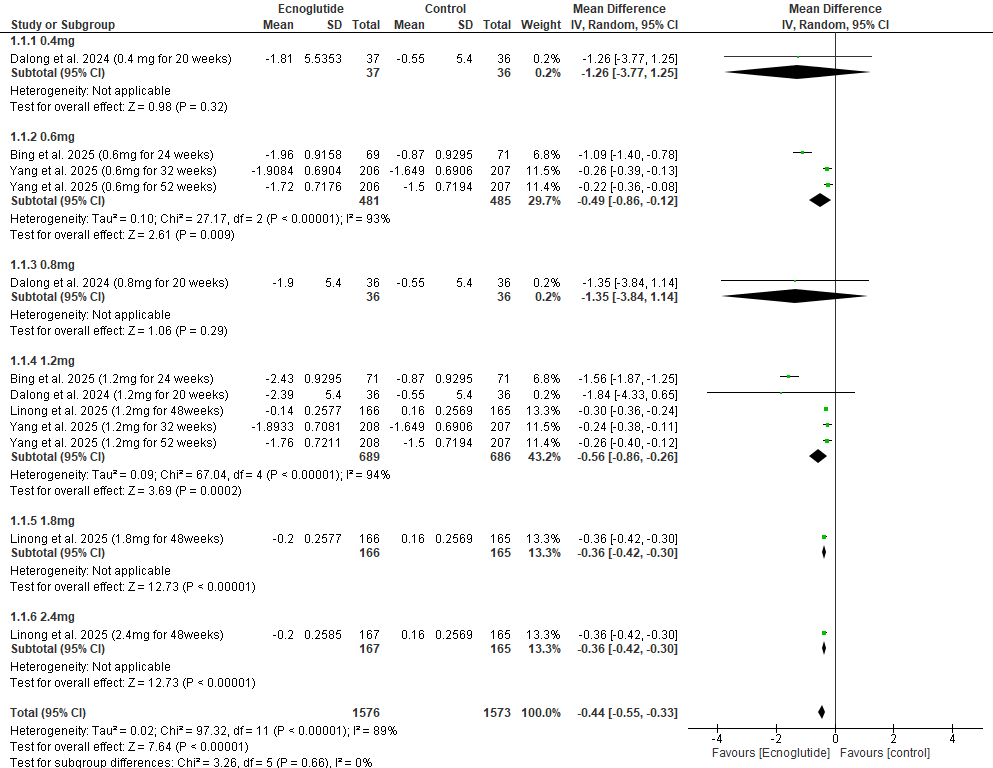
**

**Supplementary Figure 3.2: HbA1c, mmol/mol**
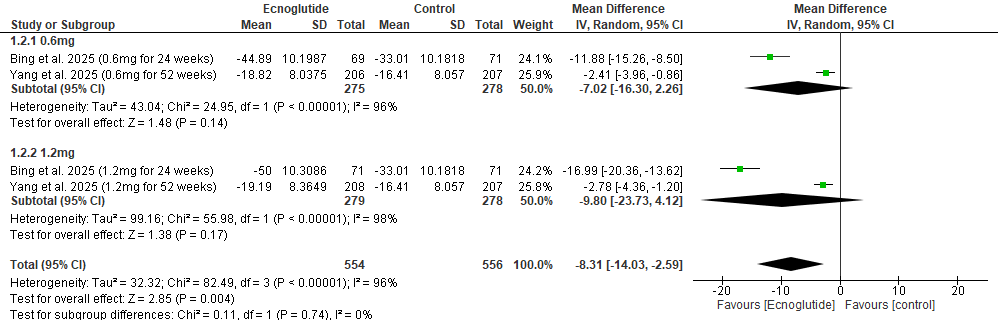


**Supplementary Figure 3.3: Fasting plasma glucose, mmol/L**
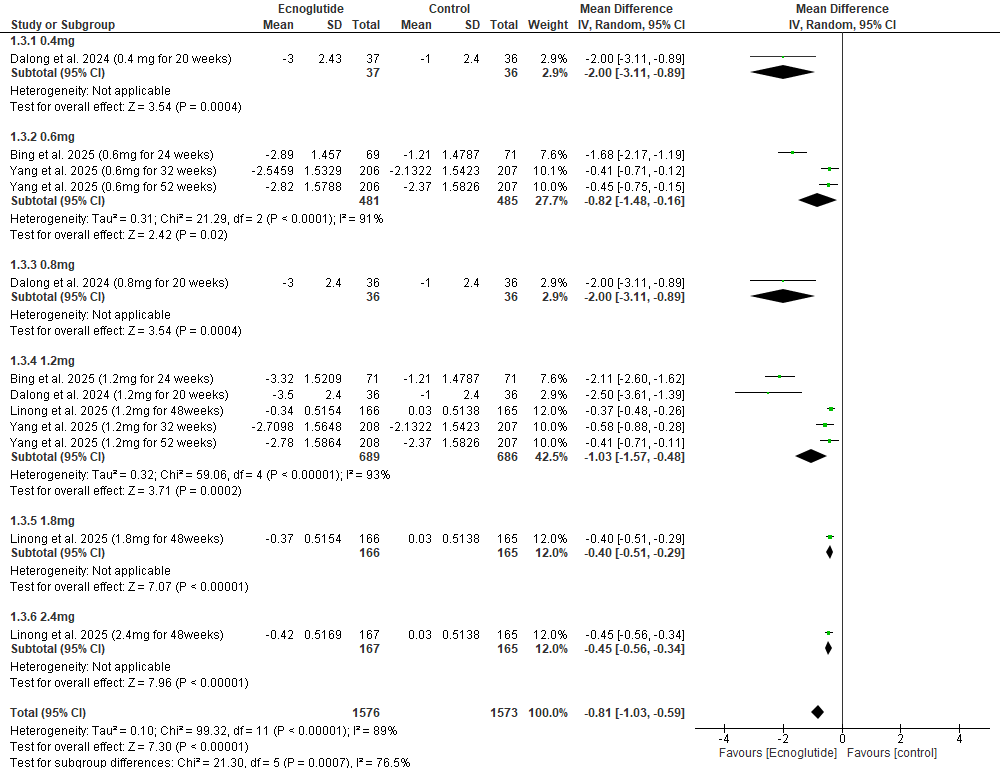


**Supplementary Figure 3.4: 2 h post-prandial blood glucose, mmol/L**
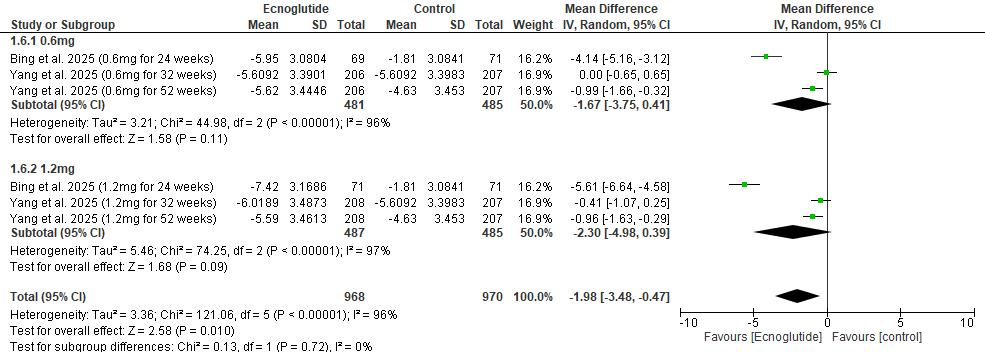


**Supplementary Figure 3.5: Fasting insulin, μU/mL**
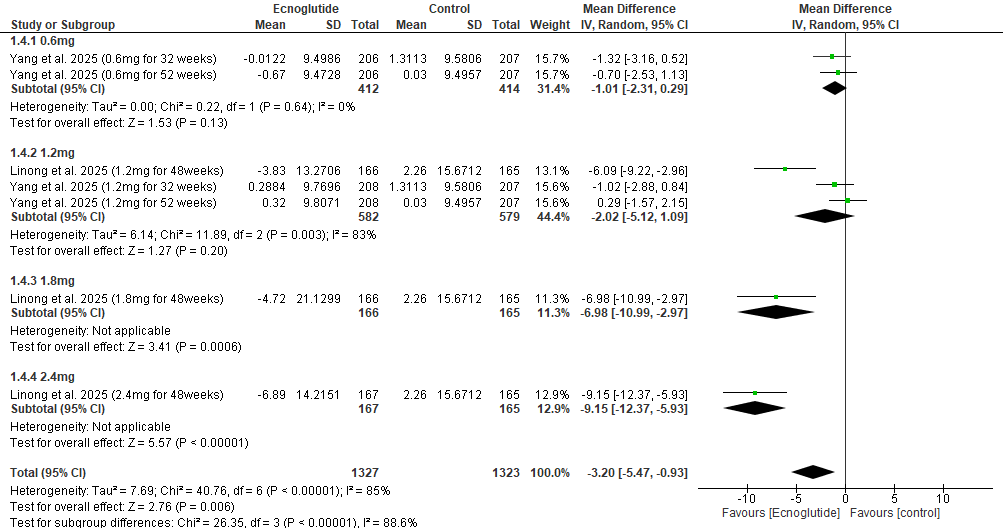


**Supplementary Figure 3.6: HOMA-IR**
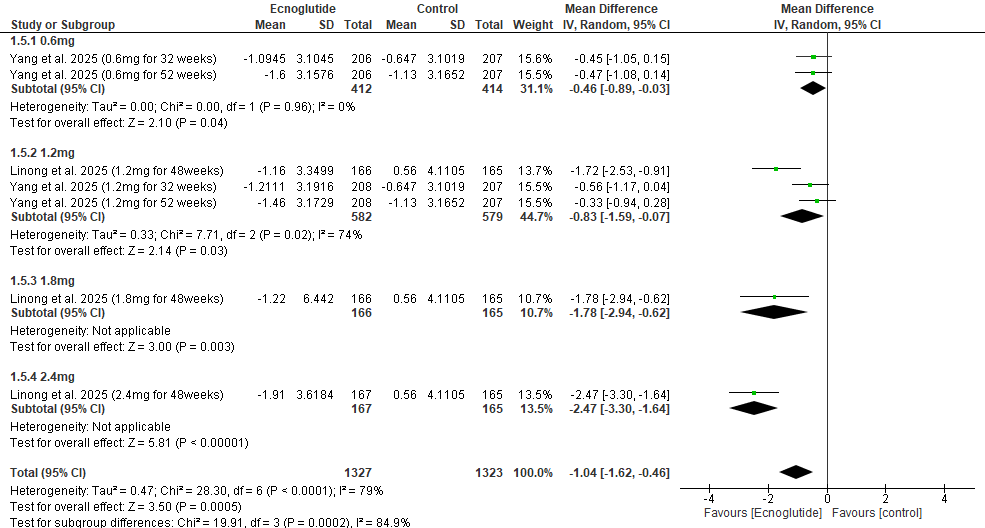


**Supplementary Figure 3.7: Bodyweight, kg**
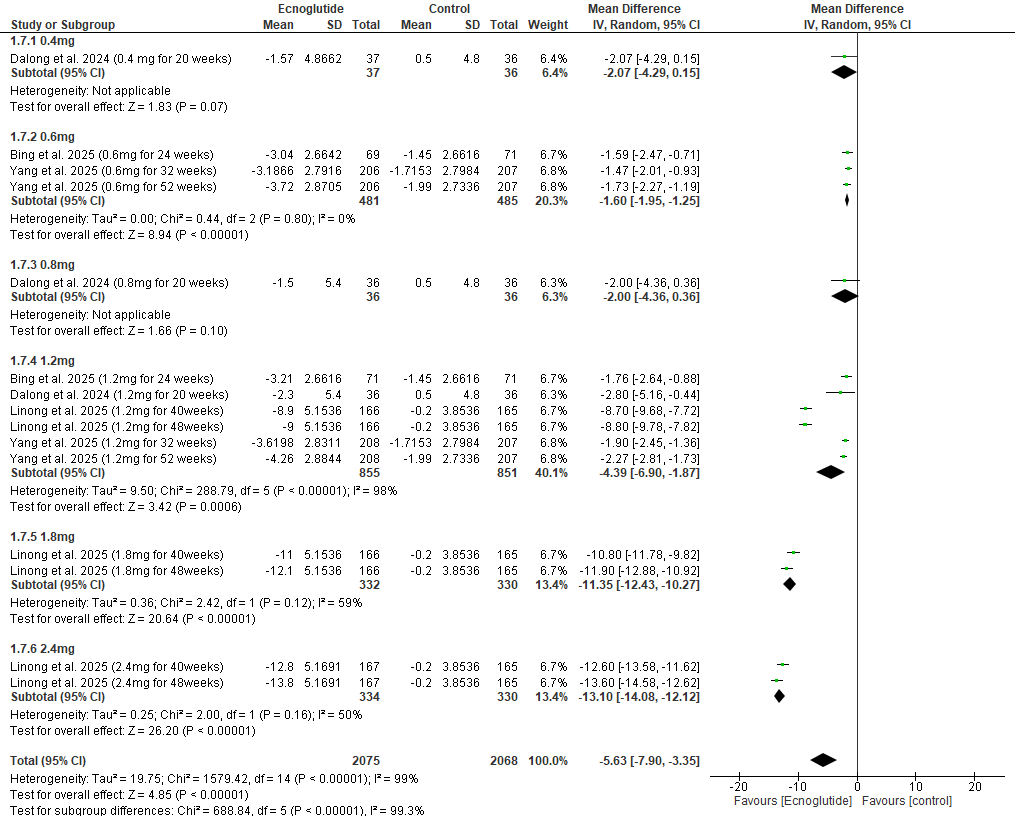


**Supplementary Figure 3.8: Percentage change in bodyweight**
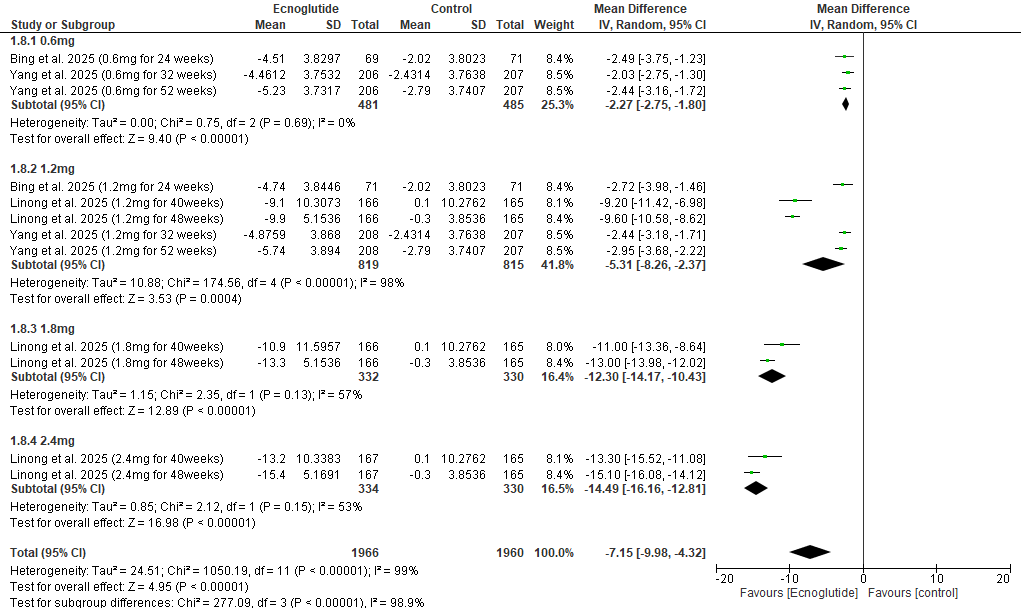


**Supplementary Figure 3.9: BMI, kg/m²**
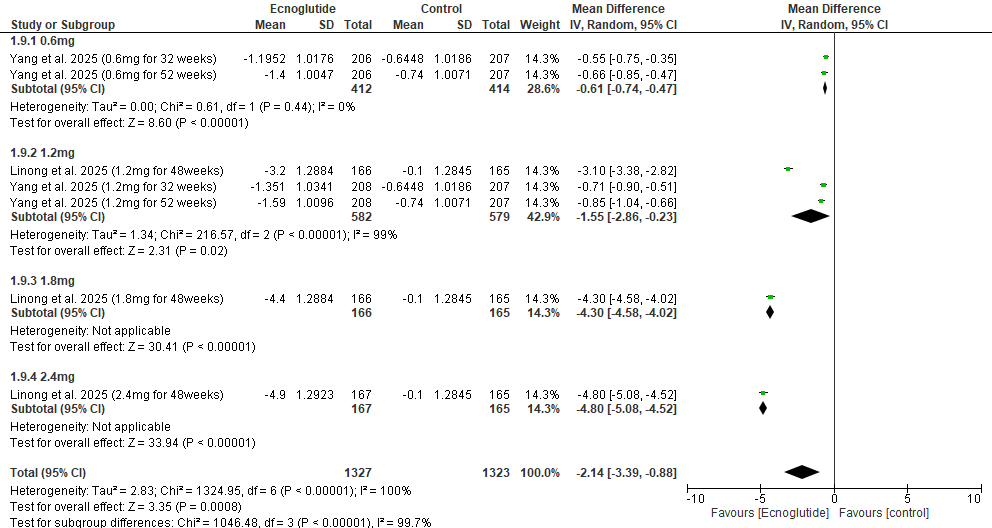


**Supplementary Figure 3.10: Waist circumference, cm**
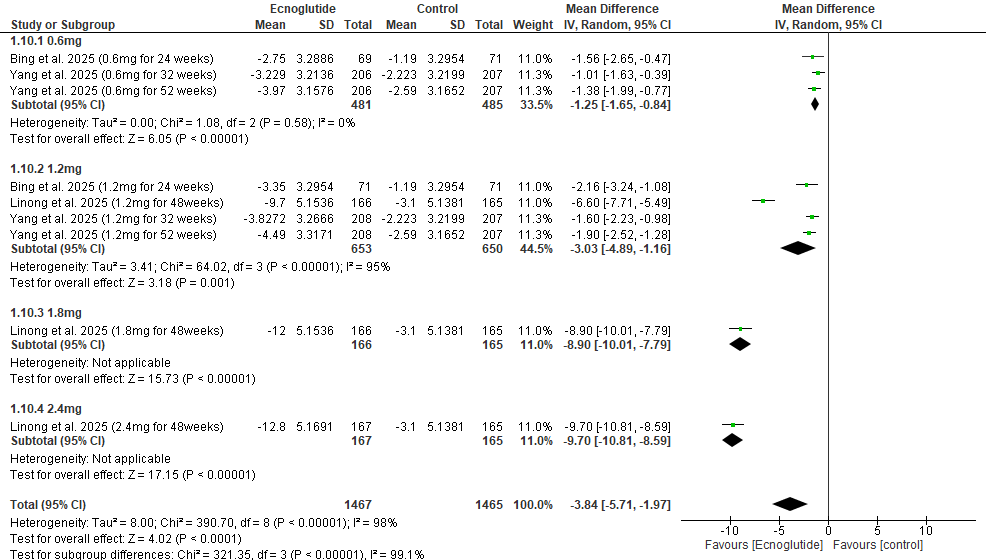


**Supplementary Figure 3.11: Hip circumference, cm**
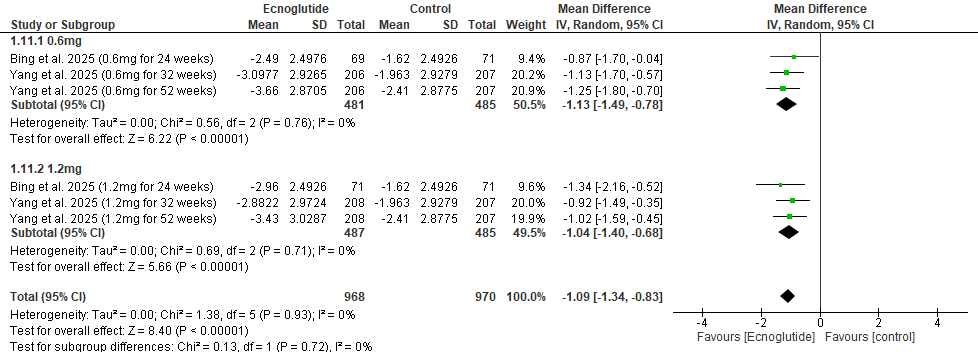


**Supplementary Figure 3.12: Percentage change from baseline in total cholesterol**
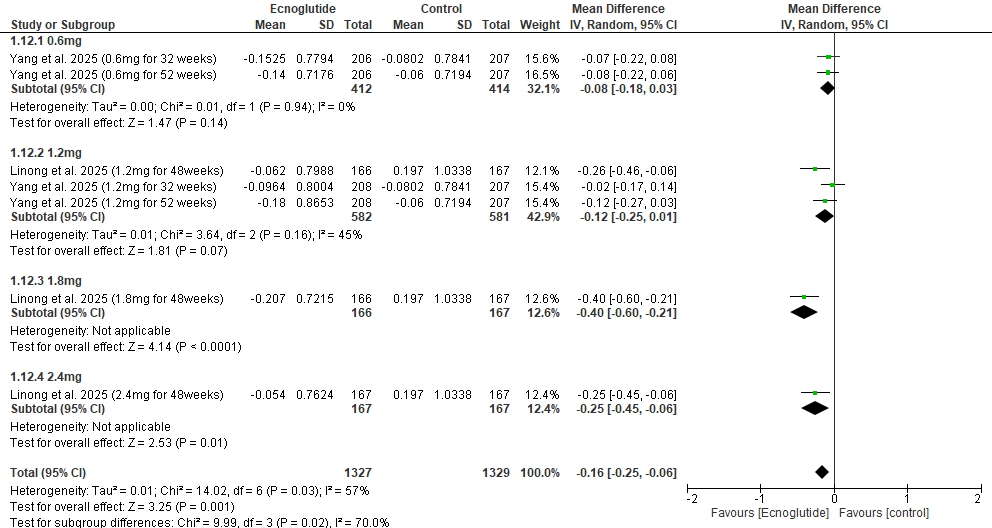


**Supplementary Figure 3.13: Percentage change from baseline in LDL-cholesterol**
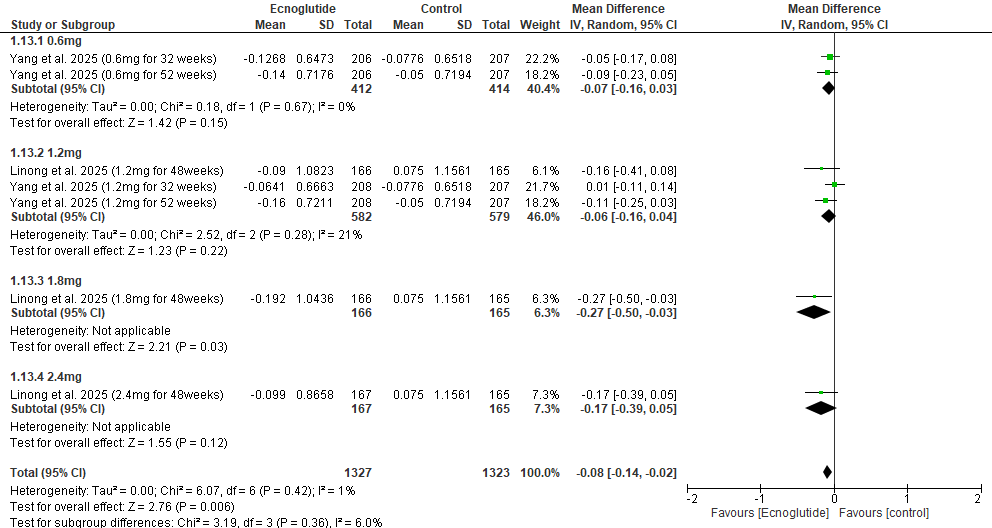


**Supplementary Figure 3.14: Percentage change from baseline in HDL-cholesterol**
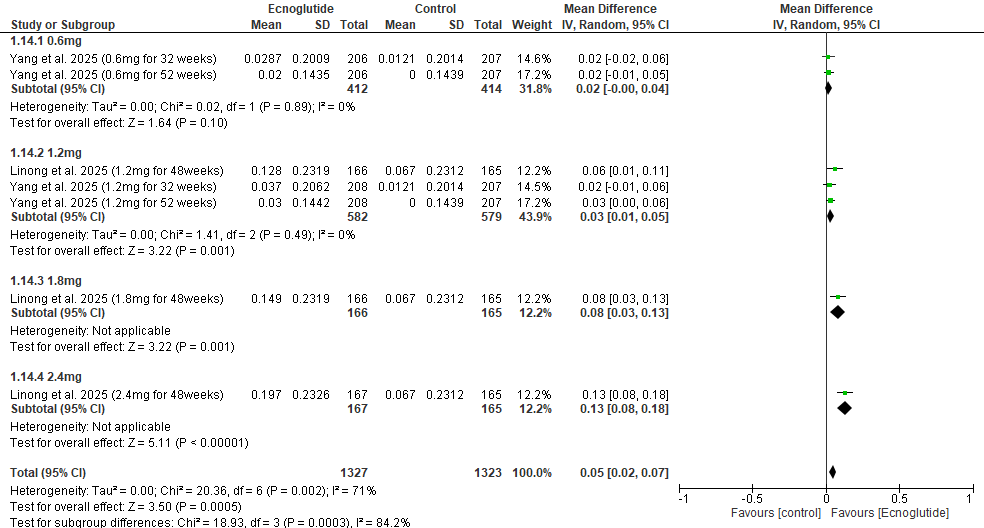


**Supplementary Figure 3.15: Triglycerides, mmol/L**
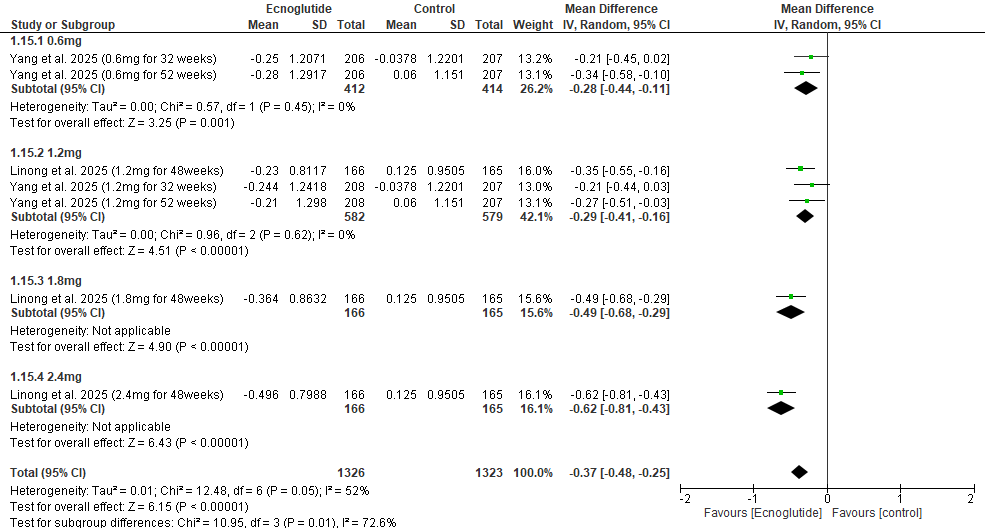


**Supplementary Figure 3.16: Alanine aminotransferase, U/L**
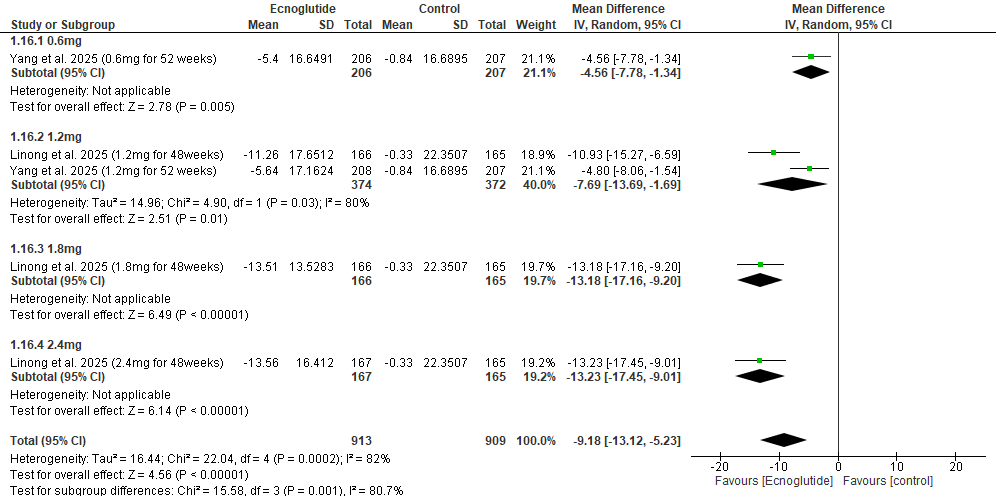


**Supplementary Figure 3.17: Aspartate aminotransferase, U/L**
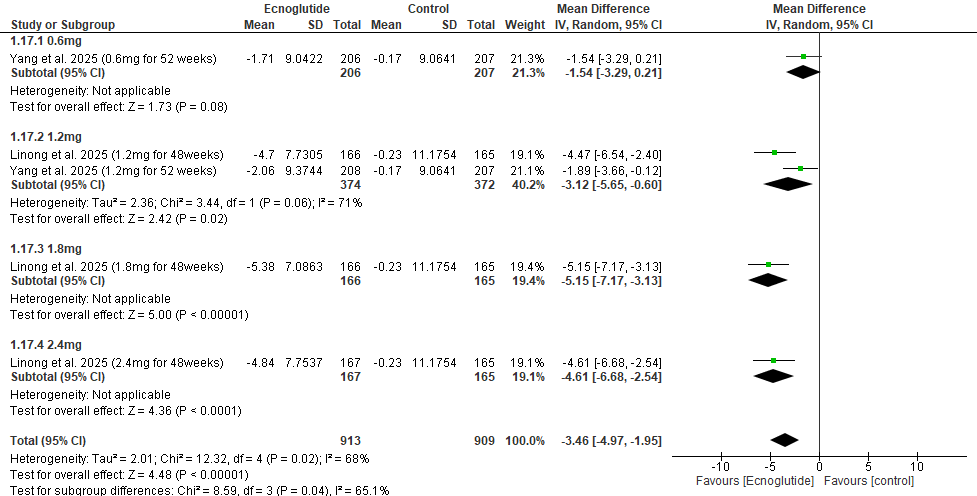


**Forest Plot of Subgroup Analysis by Follow-up Duration**

**Supplementary Figure 4.1: HbA1C %**
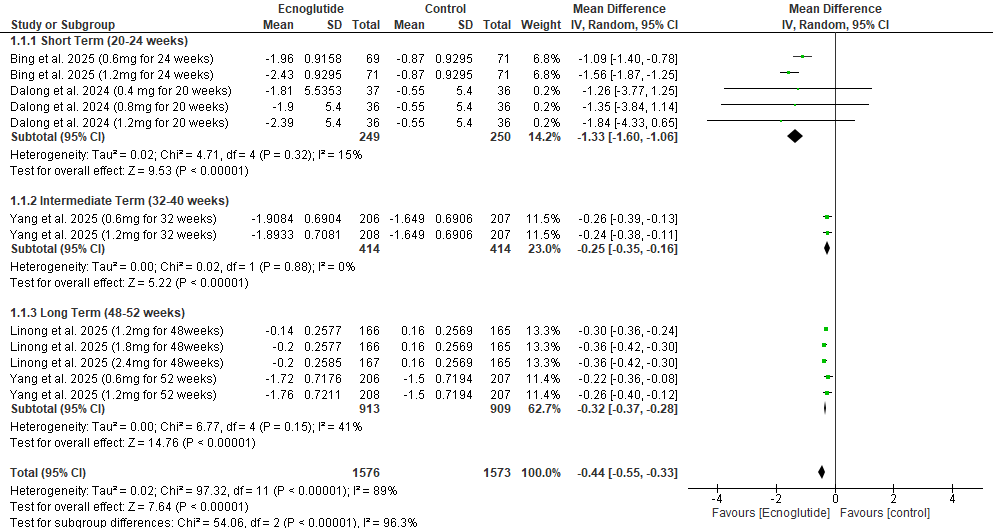


**Supplementary Figure 4.2: HbA1c, mmol/mol**
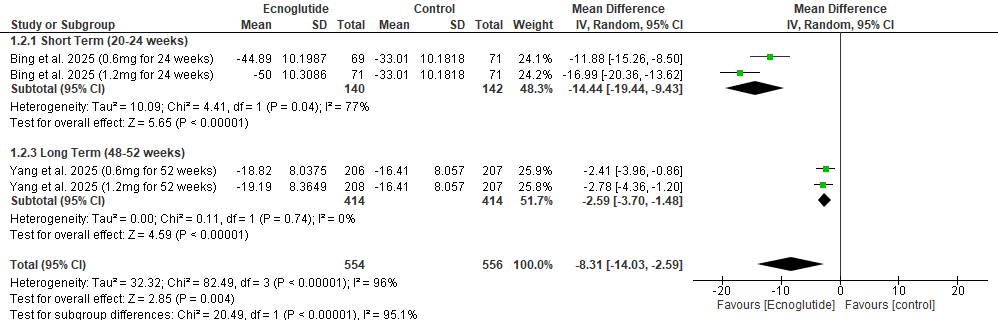


**Supplementary Figure 4.3: Fasting plasma glucose, mmol/L**
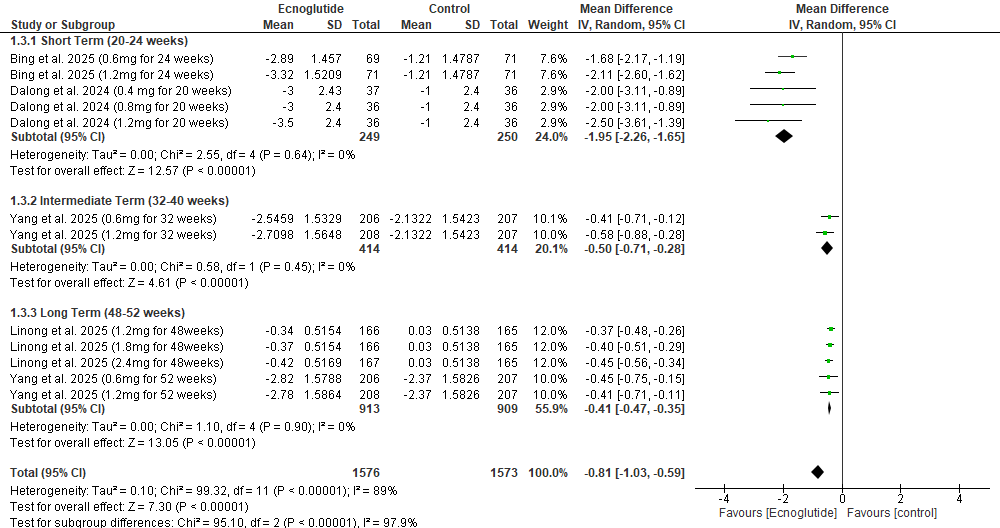


**Supplementary Figure 4.4: 2 h post-prandial blood glucose, mmol/L**
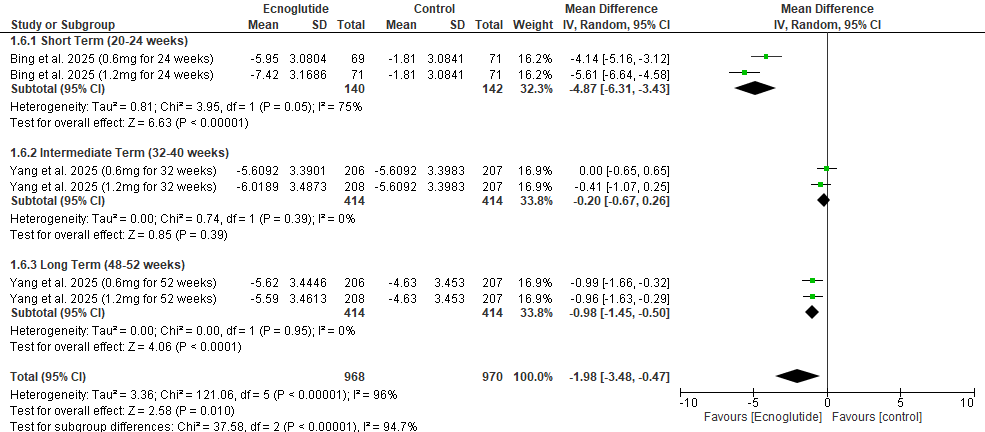


**Supplementary Figure 4.5: Fasting insulin, μU/mL**
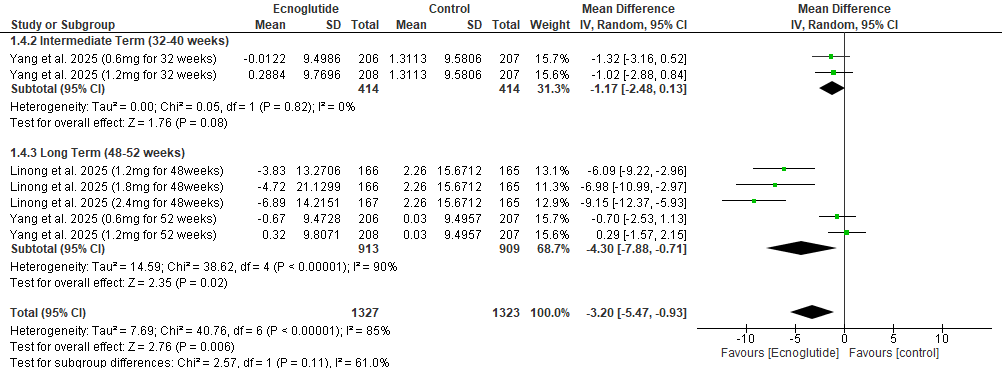


**Supplementary Figure 4.6: HOMA-IR**
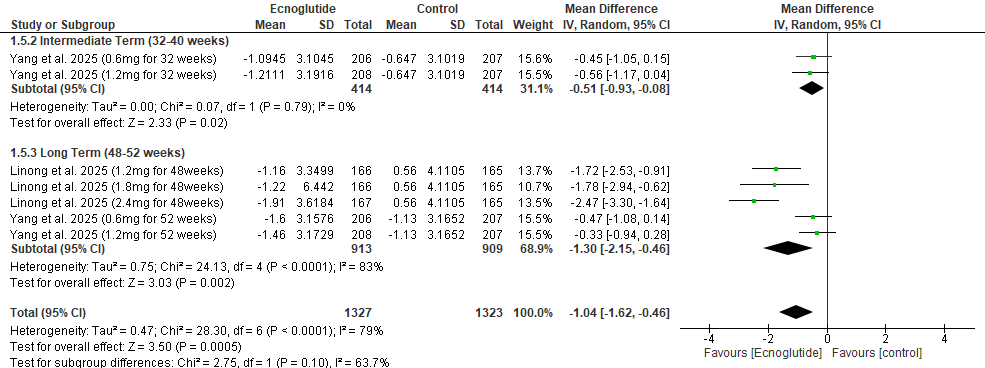


**Supplementary Figure 4.7: Bodyweight, kg**
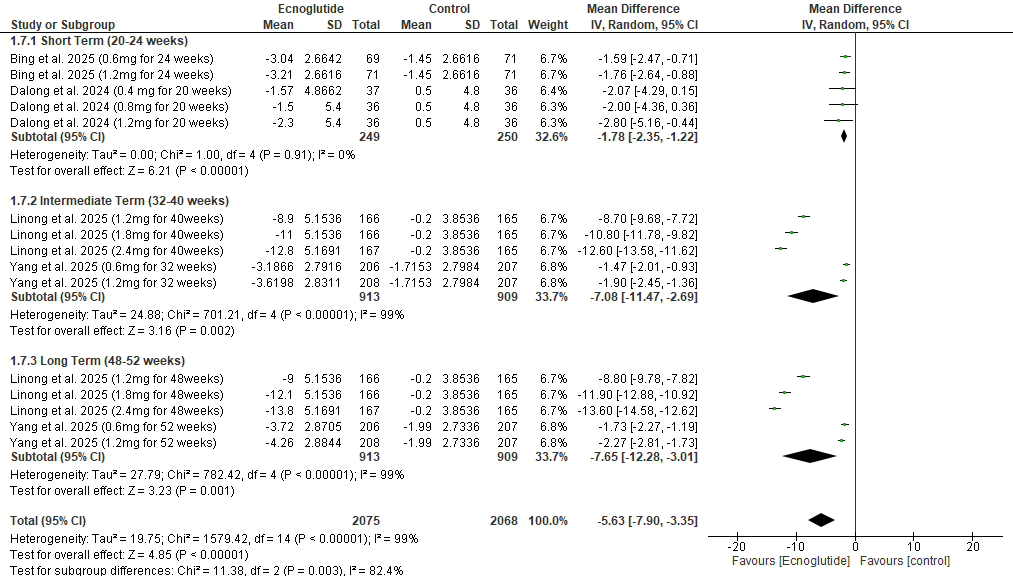


**Supplementary Figure 4.8: Percentage change in bodyweight**
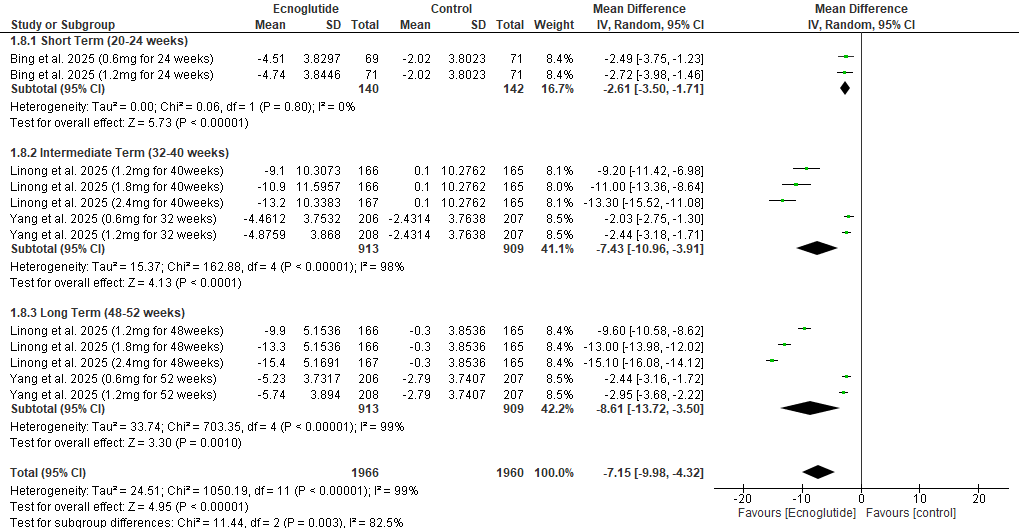


**Supplementary Figure 4.9: BMI, kg/m²**
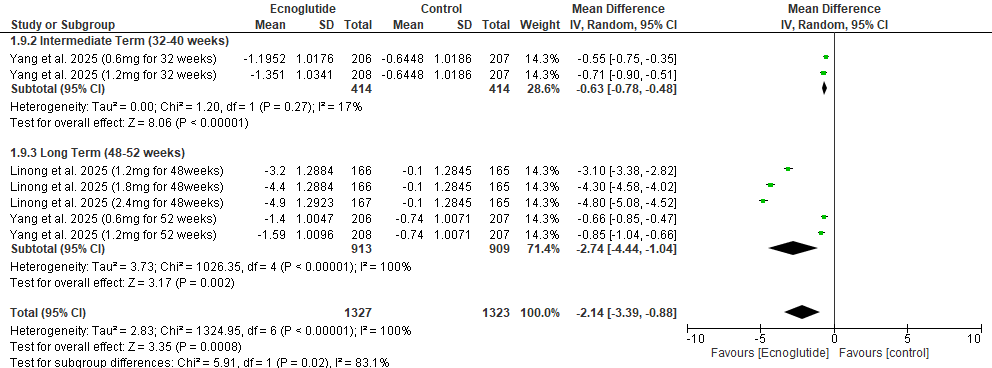
**Supplementary Figure 4.10: Waist circumference, cm**
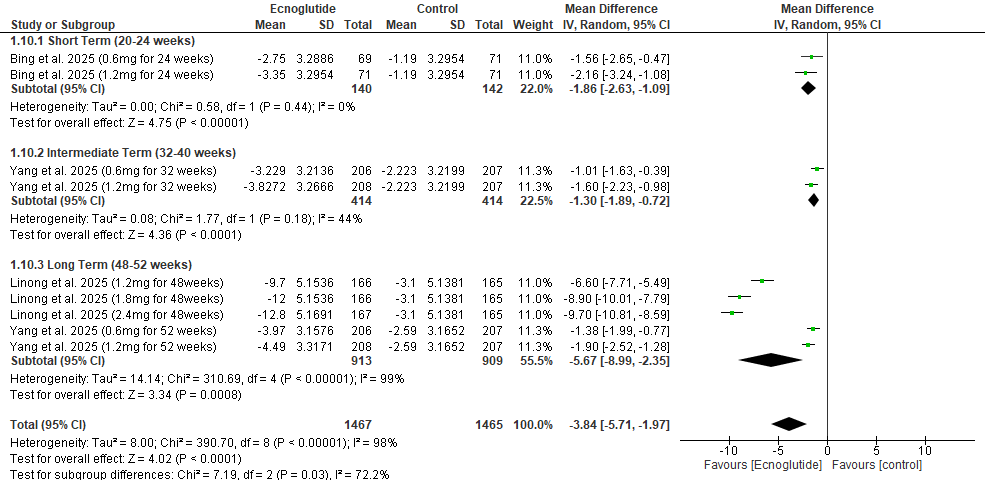


**Supplementary Figure 4.11: Hip circumference, cm**
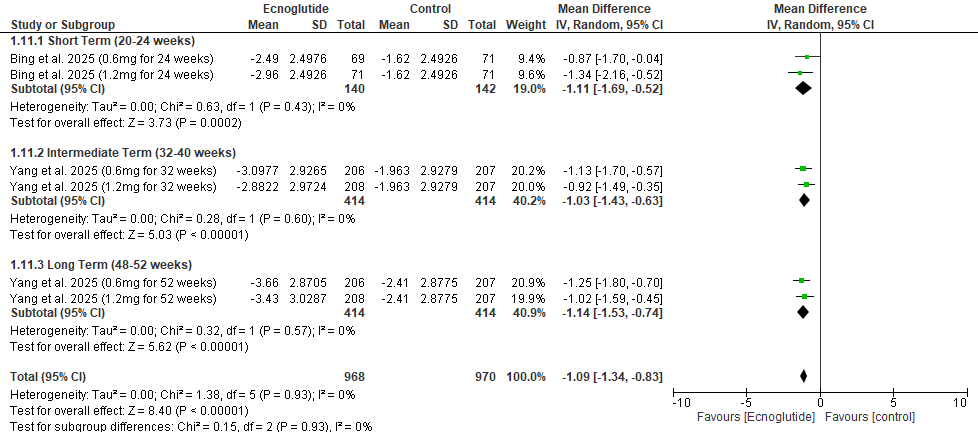


**Supplementary Figure 4.12: Percentage change from baseline in total cholesterol**
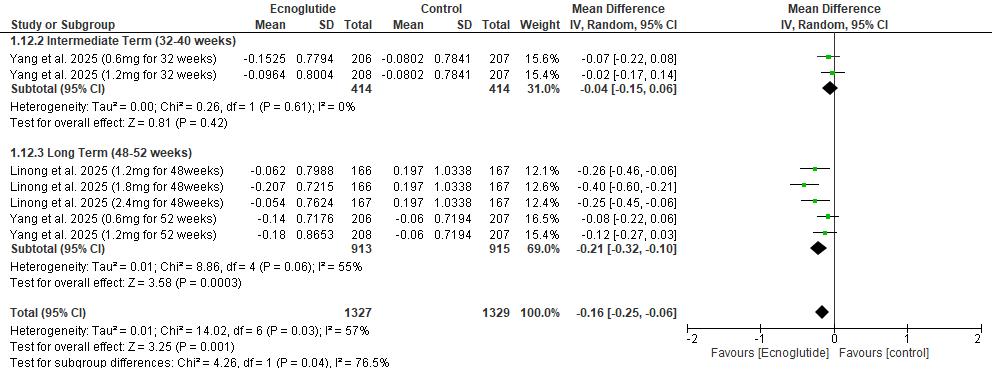


**Supplementary Figure 4.13: Percentage change from baseline in LDL-cholesterol**
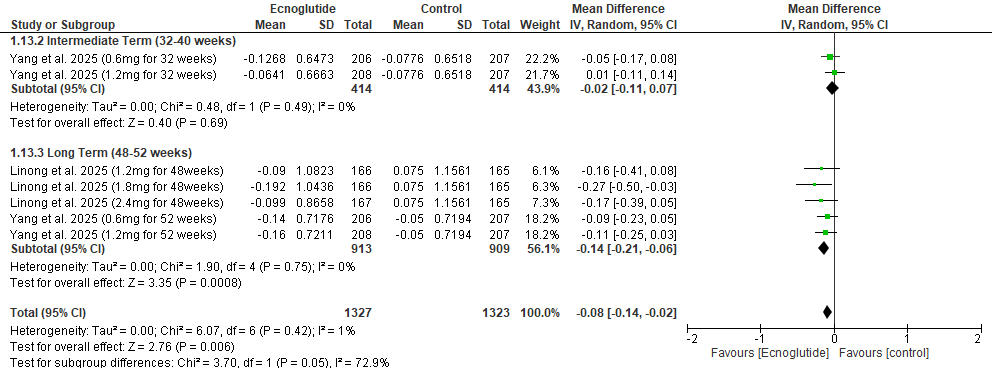


**Supplementary Figure 4.14: Percentage change from baseline in HDL-cholesterol**
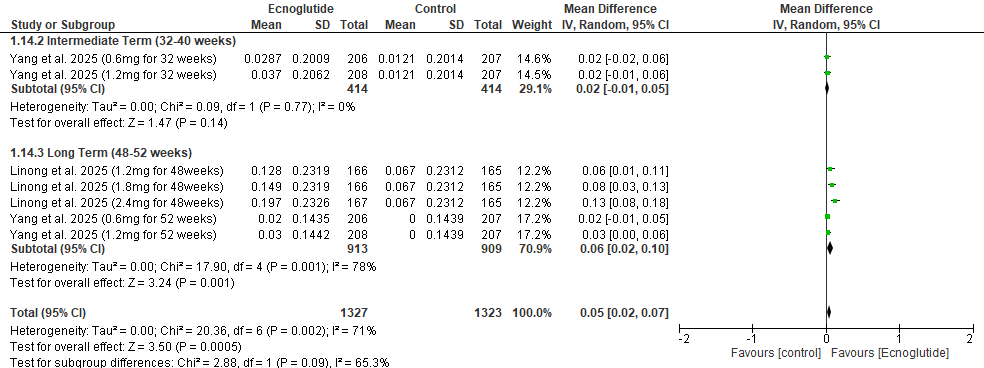


**Supplementary Figure 4.15: Triglycerides, mmol/L**
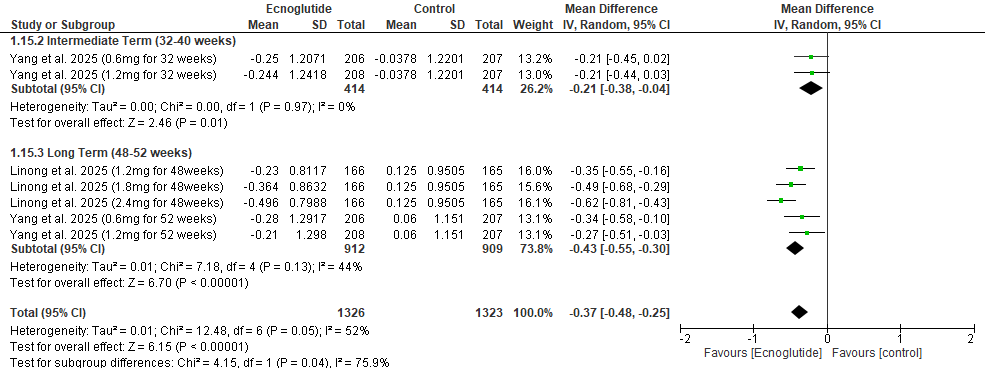

Supplement: Supplementary file 1 — Table S1: Search strategy. Table S2: Risk of bias assessment. Table S3: GRADE table for outcomes. Figure S1: 1: 2‐h post‐prandial blood glucose, mmol/L. Figure S1: 2: 7‐point SMBG, mmol/L. Figure S1: 3: Fasting insulin, μU/mL. Figure S1: 4: HOMA‐IR. Figure S1: 5: Absolute change in Bodyweight, kg. Figure S1: 6: Percentage change in bodyweight. Figure S1: 7: BMI, kg/m2. Figure S1: 8: Waist circumference, cm. Figure S1: 9: Participants with % bodyweight reduction. Figure S1: 10: Percentage change from baseline in total cholesterol. Figure S1: 11: Percentage change from baseline in LDL‐cholesterol. Figure S1: 12: Triglycerides, mmol/L. Figure S1: 13: Percentage change from baseline in HDL‐cholesterol. Figure S1: 14: Alanine aminotransferase, U/L. Figure S1: 15: Aspartate aminotransferase, U/L. Figure S1: 16: Any adverse events. Figure S1: 17: Serious adverse events. Figure S1: 18: Treatment‐related serious adverse events. Figure S1: 19: Adverse events leading to treatment discontinuation. Figure S2: 1: HbA1C % (‐Bing et al. 2025). Figure S2: 2: HbA1c, mmol/mol (‐Bing et al. 2025). Figure S2: 3: Fasting plasma glucose, mmol/L (‐Bing et al. 2025). Figure S2: 4: 2 h post‐prandial blood glucose, mmol/L (‐Bing et al. 2025). Figure S2: 5: fasting insulin, μU/mL (‐Linong et al. 2025). Figure S2: 6: HOMA‐IR (‐Linong et al. 2025). Figure S2: 7: Bodyweight, kg (‐Linong et al. 2025). Figure S2: 8: Percentage changes in bodyweight (‐Linong et al. 2025). Figure S2: 9: BMI, kg/m2 (‐Linong et al. 2025). Figure S2: 10: Waist circumference, cm (‐Linong et al. 2025). Figure S2: 11: Percentage change from baseline in total cholesterol (‐Linong et al. 2025). Figure S2: 12: Percentage change from baseline in HDL‐cholesterol (‐Linong et al. 2025). Figure S2: 13: Triglycerides, mmol/L (‐Linong et al. 2025). Figure S2: 14: Alanine aminotransferase, U/L (‐Linong et al. 2025). Figure S2: 15: Aspartate aminotransferase, U/L (‐Linong et al. 2025) Forest Plot of Subgroup Analysis by Dosage. Figure [file EDM2-9-e70217-s001.docx]
